# A Phase 2b Randomised Trial of the Candidate Malaria Vaccines FP9 ME-TRAP and MVA ME-TRAP among Children in Kenya

**DOI:** 10.1371/journal.pctr.0010029

**Published:** 2006-10-20

**Authors:** Philip Bejon, Jedidah Mwacharo, Oscar Kai, Tabitha Mwangi, Paul Milligan, Stephen Todryk, Sheila Keating, Trudie Lang, Brett Lowe, Caroline Gikonyo, Catherine Molyneux, Greg Fegan, Sarah C Gilbert, Norbert Peshu, Kevin Marsh, Adrian V. S Hill

**Affiliations:** 1 Kenya Medical Research Institute, Centre for Geographical Medicine Research (Coast), Kilifi, Kenya; 2 Centre for Clinical Vaccinology and Tropical Medicine, University of Oxford, Oxford, United Kingdom; 3 London School of Hygiene and Tropical Medicine, London, United Kingdom; 4 Wellcome Trust Centre for Human Genetics, University of Oxford, Oxford, United Kingdom; 5 Nuffield Department of Clinical Medicine, Oxford University, John Radcliffe Hospital, Oxford, United Kingdom

## Abstract

**Objective:**

The objective was to measure the efficacy of the vaccination regimen FFM ME-TRAP in preventing episodes of clinical malaria among children in a malaria endemic area. FFM ME-TRAP is sequential immunisation with two attenuated poxvirus vectors (FP9 and modified vaccinia virus Ankara), which both deliver the pre-erythrocytic malaria antigen construct multiple epitope–thrombospondin-related adhesion protein (ME-TRAP).

**Design:**

The trial was randomised and double-blinded.

**Setting:**

The setting was a rural, malaria-endemic area of coastal Kenya.

**Participants:**

We vaccinated 405 healthy 1- to 6-year-old children.

**Interventions:**

Participants were randomised to vaccination with either FFM ME-TRAP or control (rabies vaccine).

**Outcome Measures:**

Following antimalarial drug treatment children were seen weekly and whenever they were unwell during nine months of monitoring. The axillary temperature was measured, and blood films taken when febrile. The primary analysis was time to a parasitaemia of over 2,500 parasites/μl.

**Results:**

The regime was moderately immunogenic, but the magnitude of T cell responses was lower than in previous studies. In intention to treat (ITT) analysis, time to first episode was shorter in the FFM ME-TRAP group. The cumulative incidence of febrile malaria was 52/190 (27%) for FFM ME-TRAP and 40/197 (20%) among controls (hazard ratio = 1.52). This was not statistically significant (95% confidence interval [CI] 1.0–2.3; *p* = 0.14 by log-rank). A group of 346 children were vaccinated according to protocol (ATP). Among these children, the hazard ratio was 1.3 (95% CI 0.8–2.1; *p* = 0.55 by log-rank). When multiple malaria episodes were included in the analyses, the incidence rate ratios were 1.6 (95% CI 1.1–2.3); *p* = 0.017 for ITT, and 1.4 (95% CI 0.9–2.1); *p* = 0.16 for ATP. Haemoglobin and parasitaemia in cross-sectional surveys at 3 and 9 mo did not differ by treatment group. Among children vaccinated with FFM ME-TRAP, there was no correlation between immunogenicity and malaria incidence.

**Conclusions:**

No protection was induced against febrile malaria by this vaccine regimen. Future field studies will require vaccinations with stronger immunogenicity in children living in malarious areas.

## Editorial Commentary

**Background:** Malaria kills over a million people a year worldwide, and young children in sub-Saharan are particularly at risk. Cheap, safe, and effective vaccines are needed. One strategy involves a double-vaccination process. This approach (termed “prime-boost”) uses two different delivery methods to transmit the same antigen (part of a protein from the malaria parasite that can trigger an immune response). The two-step vaccination is designed to achieve a greater immune response than with just one vaccination. One research group, based in Oxford in the UK, is using an antigen called “ME-TRAP,” which is delivered using first a strain of modified fowlpox virus (called FP9), then a weakened vaccinia virus (called MVA). Previous studies done in adult UK volunteers have been promising, achieving an immune response and some protection against malaria when volunteers were deliberately infected. However, this approach has not been tested in the group most in need of a vaccine—young African children. Therefore a field trial was conducted among 405 healthy children aged 1–6 years, in a region of Kenya with year-round malaria transmission. Children were randomized to receive either the sequence of vaccines delivering ME-TRAP or to receive a rabies vaccine (as control, but which still gives the children some benefit for taking part in the trial). The children were followed up for nine months, and the primary aim of the trial was to compare the occurrence of clinical malaria (fever combined with malaria parasites in the blood) in the two groups.

**What this trial shows:** In the 387 children receiving vaccine and having at least one follow-up visit the vaccine did produce an immune response; however, this immune response did not seem to be protective, as the occurrence of malaria was slightly higher in the group receiving the candidate vaccine—although this difference was not statistically significant. Safety data were also collected; the number and severity of adverse events were similar between volunteers receiving the rabies vaccine and those receiving the candidate malaria vaccine, and any serious events were not judged to be linked to the vaccines by the trial's data safety monitoring board.

**Strengths and limitations:** The methods used in the trial were robust, using appropriate randomization procedures and blinding of participants and researchers. Outcome measures (clinical malaria, defined as fever together with parasites in the blood over 2,500/microliter) were clinically relevant. In order to detect cases of malaria in vaccinated children, health workers visited children weekly, and children with a temperature over 37.5 °C were tested for parasites in the blood. (In between the weekly visits, self-report and referral for assessment also allowed detection of cases.) This process of active detection of malaria cases (as opposed to obtaining data on clinical malaria only from self-report or referral) enables a smaller sample size to be used in the trial, but it is not clear whether this approach is more or less specific at picking up malaria cases than are passive methods. The researchers aimed to ensure that their case detection methods were specific; for children with normal temperature, but reported by their parents as feverish, parasite tests were done only if subsequent temperature readings were high.

**Contribution to the evidence:** Previous studies of ME-TRAP using the FP9 and MVA vectors have shown the candidate vaccine is safe and induces a strong immune response. The safety result was also supported by the findings of the current trial, conducted in young Kenyan children, but a 5-fold lower immune response was found compared to previous studies. The trial showed that this weak immune response was not effective at preventing clinical cases of malaria in this group of children, although it is not clear why the immune response was lower than expected.

## INTRODUCTION

More than 1 million people die each year from malaria, and this number is likely to increase [1]. A vaccine is urgently needed. There is evidence that T cells are protective against malaria in animal models [2], field studies [3], and following immunization with irradiated sporozoites [4]. This evidence has prompted the development of a heterologous prime-boost strategy to induce T cell responses against pre-erythrocytic stages of parasite development [5]. The prime-boost strategy uses two different vectors to deliver a common antigen construct, which achieves an expansion of T cells reactive to the common antigen, rather than to the vectors used [6].

Prime-boost vaccination with FP9 (an attenuated fowlpox virus) then with modified vaccinia virus Ankara (MVA), both recombinant for the pre-erythrocytic antigen construct (the multiple-epitope string and thombospondin-related adhesion protein, ME-TRAP [7]) was safe, immunogenic, and partially protective in malaria-naïve adults exposed to experimental challenge [8]. After controlled bites from Plasmodium falciparum-infected mosquitoes, some individuals were fully protected and vaccinees showed mean delays in time to parasitaemia, corresponding to a mean 92% reduction in malaria parasites completing pre-erythrocytic development [9]. However, prime-boost vaccination with DNA priming and MVA (delivering the ME-TRAP insert) was not protective against parasitaemia in semi-immune adults [10].

However, incidence studies in semi-immune adults are complicated by the fact that adults have greater naturally acquired immunity than do children; this natural immunity increases with age [10]. Reinfection rates are similar despite different entomological inoculation rates (EIRs) [11,12], and so such studies might then not identify vaccine-induced reductions in malaria parasites completing pre-erythrocytic development. Incidental insecticide-treated net (ITN) use was not associated with a reduced risk of parasitaemia in longitudinal follow-up studies of semi-immune adults [10,13]. Thus, a partially effective pre-erythrocytic vaccine might be more effective against febrile disease in children than against asymptomatic parasitaemia in semi-immune adults. Furthermore, FP9 priming was significantly more immunogenic and protective than DNA priming in mice [14], and more protective in nonimmune volunteers, although not significantly so (*p* = 0.3) in the small sample studied [8].

The response to vaccination was measured by two different enzyme-linked immunosorbent spot (ELISPOT) assays. An ex vivo ELISPOT assay was used to count IFN-γ-producing effector T cells after overnight incubation with antigen, and a cultured ELISPOT assay was used to measure resting memory cells [15]. These assays appear to measure different cell populations. A study of naturally acquired immunity to circumsporozoite protein demonstrated no association between cultured ELISPOT and ex vivo ELISPOT results [16]. Furthermore, the T cells induced by vaccination that are identified by cultured ELISPOT persist for at least six months after vaccination of naïve participants, despite waning numbers of cells detected by ex vivo ELISPOT [17]. The cultured ELISPOT response to TRAP after prime boost and to the circumsporozoite protein after the malaria vaccine RTS,S in various adjuvants was associated with protection following sporozoite challenge [17,18].

Immunogenicity and safety data were acquired for adults and then children in Phase 1 studies in Kenya before Phase 2b studies were undertaken, including the FFM ME-TRAP regimen (i.e., two sequential FP9 vaccinations followed by MVA, where both vectors deliver ME-TRAP) that is used the present study [19–21]. Local and systemic reactogenicity was mild. Immunogenicity was lower than, but comparable to, that seen among partially protected volunteers in sporozoite challenge studies.

The primary aim of this Phase 2b trial was to evaluate safety, immunogenicity, and efficacy of the vaccination in the target population (i.e., children in a malaria-endemic area), using mild febrile malaria as an endpoint. To detect cases, we used active weekly monitoring for fever, and made blood films from febrile children. Field workers lived in the community, and were easily accessed by parents when fever occurred between regular visits.

## METHODS

### Study Design

The study was randomised, controlled, and double blind. Ethical approval was obtained from the Kenyan Medical Research Institute National Ethics Committee, the Central Oxford Research Ethics Committee, and the London School of Hygiene and Tropical Medicine Ethics Committee. An independent Data Safety Monitoring Board and a Local Safety Monitor were appointed. The Data Safety Monitoring Board reviewed all serious adverse events as they occurred, approved the selection of vaccine dose based on previous Phase 1 studies, and reviewed the analysis plan. Research was conducted in accordance with the Helsinki Declaration of 1975 (revised 1983). The trial was conducted according to Good Clinical Practice. Oxford University, as trial sponsor, arranged external monitoring and oversaw the conduct of the trial.

At least one dose of vaccine was given to 405 children. Blood tests for immunology, safety, and cross-sectional assessments of malaria parasitaemia were conducted prevaccination, at screening, 1 wk after the third vaccination, then at 3 mo and at 9 mo. Monitoring for solicited adverse events was conducted for 1 wk after each vaccination. Unsolicited adverse events and episodes of malaria were monitored throughout the 1 y study duration. Children were screened in February 2005, immunised between March 2005 and May 2005, and followed up until February 2006. Monitoring is continuing in a further study.

### Participants

The participating children were aged 1–6 y (inclusive), healthy, and resident in the study area. After a series of public meetings and individual discussions, a screening date was set at which study information was repeated, and consent was sought before proceeding. Participants were not immunized until at least one week after their parents signed consent, to allow the parents time to consider their decision.

Children were screened by history, examination, and blood tests (complete blood count, creatinine, and alanine transaminase). Those with clinically significant illness were excluded. Clinically evident immunosuppression was one of the criteria for exclusion, but no children were excluded on this basis. HIV testing was not conducted. Abnormal alanine transaminase or creatinine levels were exclusion criteria, but anaemia without clinically significant signs and symptoms was not (although iron supplementation was given to the 27 children with haemoglobin > 8 g/dl). Recent blood transfusions (within the previous 2 mo), current participation in another clinical trial, or receipt of another experimental vaccine were also exclusion criteria. Eligible children were invited to attend vaccination in the order in which they were screened.

### Location

The study was carried out in Junju sublocation in Kilifi District, on the Kenyan coast. Junju contains a group of five closely related villages within the Chonyi area of Kilifi district. Junju lies between Kilifi and Mombasa, 14 km inland from the coastal road. Kilifi is malaria-endemic. There are two high-transmission seasons, but low-level transmission continues all year-round [22]. The transmission intensity is 22–53 infective bites per year [23]. Junju is served by a local government dispensary, which has an active ITN distribution programme. Inpatient care is available at Kilifi District Hospital.

### Interventions

The antigen insert used in the vaccine was TRAP, joined to a multiple epitope (ME) string from six P. falciparum pre-erythrocytic antigens [7]. The ME string contains 14 pre-erythrocytic MHC class I epitopes from six P. falciparum pre-erythrocytic antigens, three class II epitopes, two pre-erythrocytic B cell epitopes and pb9 (a P. berghei T-cell epitope that allows pre-clinical potency and stability testing).

The vectors used were an attenuated fowlpox virus, FP9, and MVA. Recombinant vaccine stock was supplied to contract manufacturer IDT (Rosslau, Germany), who produced clinical lots under Good Manufacturing Practice conditions. Single batches of FP9 ME-TRAP and MVA ME-TRAP were used throughout (both batch #051204).

The trial vaccination regimen was two sequential FP9 ME-TRAP vaccinations (5 × 10^7^ plaque-forming units) followed by MVA ME-TRAP vaccination (1.5 × 10^8^ plaque-forming units), given intradermally. The control was rabies vaccine (Aventis Pasteur, WISTAR strain), administered according to the same timings. Rabies was also given intradermally, at 0.25 IU. Vaccinations were spaced four weeks apart (acceptable range three to five weeks).

Vaccines were shipped to Kenya on dry ice with logged temperature monitoring, stored at −80 °C, and transported to the field in cool boxes for use within six hours. Vaccines were given intradermally over the deltoid area of the nondominant arm, using a 27-gauge needle to raise a visible intradermal bleb. Volunteers were observed for one hour with resuscitation facilities available for advanced life support. Children received vitamin A supplements with each vaccination, as per Government of Kenya guidelines, and parents were supplied with two doses of paracetamol syrup for use if the child developed fever at night.

### Objectives

The objectives of this Phase 2b trial were to evaluate safety, immunogenicity, and efficacy of the vaccination in the target population (i.e., children in a malaria-endemic area), using mild febrile malaria as an endpoint.

### Outcomes

#### Efficacy (malaria episodes).

The primary endpoint was a clinical episode of malaria, defined as an axillary temperature greater than 37.5 °C, with a P. falciparum parasitaemia greater than 2,500 parasites/μl. The presence of any falciparum parasitaemia with fever above 37.5 °C was a secondary endpoint.

Asymptomatic parasitaemia is common in children under endemic conditions (70% of children in this study). If a child with asymptomatic parasitaemia is evaluated during an unrelated viral illness, they will be wrongly ascertained as a case of febrile malaria [24]. An endpoint with low specificity will lead to an incorrect estimate of efficacy [25]. Since active surveillance was used, the specificity of the endpoint was maximized in three ways. A previously defined threshold parasitaemia was used for the diagnosis of malaria [22], all children received curative treatment for malaria before monitoring began, and only children in whom fever was confirmed by measurement (rather than simply by verbal report) had blood films made.

The curative treatment was given one week after the final vaccination, using seven days of directly observed dihydroartemisinin monotherapy (2 mg/kg on the first day, followed by 1 mg/kg for 6 d). Blood films were taken to confirm that children were parasite-negative one week after the end of treatment.

Children were then visited every week by field workers. The mother was asked whether she thought the child was hot, and the axillary temperature was also measured. When the temperature was greater than 37.5 °C, a blood film was made and a rapid near-patient test for malaria conducted. The blood film was reviewed within 24–48 h, but treatment decisions were based on the rapid test result.

When the mother reported that the child was hot, but an objectively elevated temperature was not identified, blood films and rapid testing was not performed, but the field worker returned to the child three times in the next 24 h. Rapid testing and blood films were performed if the child's temperature was elevated on any of these visits. Parents could bring their children for assessment between regular weekly visits if they thought the child had developed fever, and the child was assessed as above. Field workers were recruited from the villages in which the study was conducted, and so were readily accessible to the parents. Treatment for episodes of malaria was with the Government of Kenya-recommended first-line treatment, artemether-lumefantrine.

During blood tests for safety and T cell response, blood films were made on all children. The results of this testing was not available for several weeks, during which monitoring of febrile illnesses continued. Asymptomatic parasitaemia was therefore not treated unless the child developed a fever. All blood films (from well children and febrile children) were counted in duplicate by two microscopists, and a third count was conducted if they were discrepant.

#### Immunogenicity by ELISPOT.

Peripheral blood mononuclear cells (PBMCs) were separated at screening, one week after the last vaccination, then at three months and at nine months. PBMCs were incubated in RPMI media (Sigma-Aldrich, St. Louis, Missouri, United States) with 10% human AB serum. ELISPOTs used Millipore MAIP S45 plates (Millipore, Billerica, Massachusetts, United States) and MabTech antibodies (MabTech, Stockholm, Sweden) according to manufacturer's instructions. Freshly isolated PBMCs were incubated at 4 × 10^5^ cells/well in a volume of 100 μl with 10 μg/ml peptides for 18–20 h before the ELISPOT was developed. Individual 8- to 17-residue epitopes were pooled for the ME string. Peptides of 20 residues overlapped by ten residues were used for TRAP. TRAP peptides were pooled according to region. Phytohaemagglutinin (Sigma-Aldrich) was used at 20 μg/ml as positive control, and PBMCs were cultured in medium alone as negative control. Spot-forming cell numbers were counted by an ELISPOT plate reader (version 3.0; Autoimmun Diagnostika, Strassberg, Germany).

For cultured ELISPOTs, 1 × 10^6^ PBMCs were incubated in 0.5 ml of 10 μg/ml/peptide of pooled TRAP and ME peptides in a 24-well plate. On days 3 and 7, 250 μl of culture supernatant was replaced with 250 μl of culture medium containing 20 IU/ml of recombinant IL-2. On day 9 the cells were washed three times and left overnight before the standard ELISPOT assay.

### Adverse Events

Field workers visited patients daily for the first three days after vaccination and at one week after vaccination. Solicited adverse events were recorded, the diameters of skin discolouration were noted, and blistering was measured. Loss of the epidermis or upper part of the dermis was described as a deroofed blister. If unsolicited adverse events were reported, these were assessed and documented by a medically qualified investigator. Blood tests for routine biochemistry (plasma alanine transaminase and creatinine) and haematology (complete blood counts) were conducted 7 d after the final vaccination, then at 3 mo and 9 mo.

### Sample Size

We expected the incidence of febrile malaria to be 50%. The study was designed to detect 35% efficacy with 80% power by enrolling 400 children. In fact malaria transmission was lower than expected (as in most of East Africa in 2005); only a 25% incidence was recorded, so the actual trial was only powered to detect a 50% efficacy.

### Randomization—Sequence Generation

The randomization sequence was generated by the trial statistician in the UK, who had no involvement in enrolment, follow-up, or assessment of participants. Eligible children were assigned a randomization number, and vaccine group was allocated using restricted randomization in blocks of ten after sorting the list of eligible individuals by age and village.

### Randomization—Allocation Concealment

Opaque randomization envelopes were prepared and sealed in the UK without the involvement of any investigators participating in enrolling or assessing children at the study site. Each child was assigned the envelope bearing his or her study number; the envelope was opened when the child attended for the first vaccination.

### Randomization—Implementation

The investigators in Kenya enrolled children. Study numbers were applied sequentially. A list of eligible children was then ordered according to age and village, and matched to the list of randomization card numbers. If children did not attend for the first vaccination, the card was then assigned to a child of similar age from the same village.

### Blinding

The nurses who administered vaccinations did not take part in any other trial-related procedure, and were subsequently based in Kilifi District Hospital rather than the trial site. They drew up vaccinations according to the instructions in the randomisation envelope, and documented the vaccination in notes that were not available to the investigators until after unblinding, after follow-up was completed for all children. The randomisation envelopes were resealed after each vaccination and stored in a locked office for reuse at subsequent vaccinations. Neither parents nor investigators were told of the vaccination allocation, and the investigators did not enter the vaccination room while vaccinations were conducted.

Intradermal poxvirus vaccinations are associated with skin discolouration and blistering [21]. These effects were unlikely to have compromised blinding, since children were routinely assessed by field workers who were unaware of an expected difference in local reactogenicity. Furthermore, 16% of intradermal rabies vaccinations in the trial were associated with skin discolouration.

### Statistical Methods

No interim analyses were planned or conducted, and the analysis plan was approved by the Data Safety Monitoring Board before unblinding. The primary analysis was a log-rank test comparing the time to the first or only episode of malaria (defined as fever with parasitaemia above 2,500/μl) between the vaccination groups, stratified by age group, ITN use, and village, for both according-to-protocol (ATP) and intention-to-treat (ITT) participants. The hazard ratio and 95% confidence interval (CI) were estimated by Cox's regression adjusted for the same covariates. Age group was a categorical variable with three levels (ages 1–2 y, 2–5 y, and 5–6 y). Village had five levels. ITN use was defined as sleeping under a treated net every night, which had fewer than three holes into which a finger could comfortably fit.

Poisson regression was used to estimate the incidence rate ratio taking into account all malaria episodes, adjusted for the same covariates. A period of 28 days after each malaria episode was deducted from the person time at risk, since individuals were assumed not to be at risk of malaria during this period. In secondary analyses, malaria was defined as fever with parasitaemia at any density. ELISPOT results were log-transformed, substituting one spot per million for negative results (half the lower limit of detection), and then Student's t-tests used to compare results. To examine the effect of ELISPOT results on episodes of malaria, the responses were divided into tertiles, and the tertile was then used as a categorical variable.

The analysis plan specified that monitoring of malaria episodes would continue for a further nine months, after which a second analysis of episodes would be conducted.

## RESULTS

### Participant Flow

A total of 530 children were screened. Eligible children were invited to attend for vaccination in the order in which they were screened. Vaccination continued until 400 children were immunised.

### Recruitment

Children were recruited over a five-week period, and all immunizations took place from March to May 2006.

### Baseline Data

The trial participants were balanced between treatment allocation groups in both demographic characteristics at baseline and progress through the trial (Table 1). Of 386 children, 275 (71%) were parasitaemic at baseline. Antimalarial treatment was given to 73 children for intercurrent febrile malaria during vaccinations. Blood films were not taken on the days of vaccination, but 179 (52%) of 374 children were parasitaemic one week following final vaccination, before receiving antimalarials at the start of monitoring.

**Table 1:**
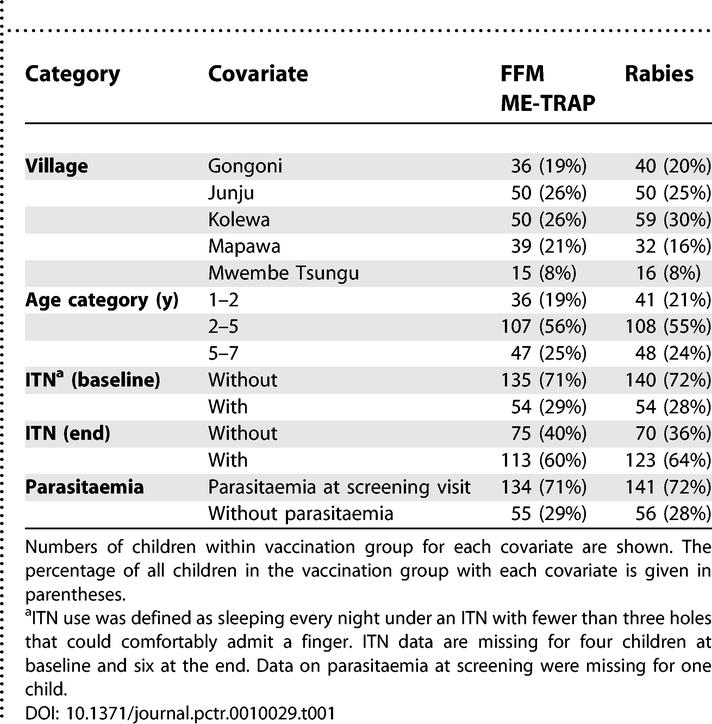
Baseline Covariates

### Numbers Analyzed

**Figure 1:**
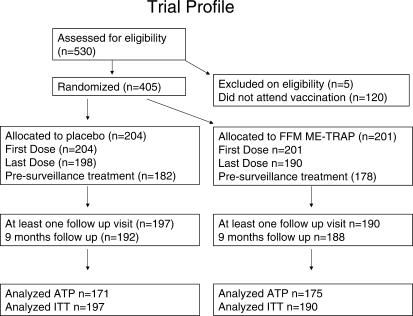
Trial Profile After screening for eligibility, parents were invited to bring their children back to the dispensary for immunisation. Children were randomised on attending for vaccination. Of the 17 children who attended for the first, but not the final, vaccination, two had moved out of the area, and parents of the remaining 15 chose not to reattend. No severe adverse events were identified in these children. Before 9 mo monitoring was complete, eight children had moved out of the area.

In total 346 children were vaccinated ATP, and an additional 41 children were included only in the ITT analyses. The distribution of ATP and ITT groups by vaccination is given in Figure 1.

### Outcomes and Estimation

#### Efficacy (primary analysis).

**Figure 2:**
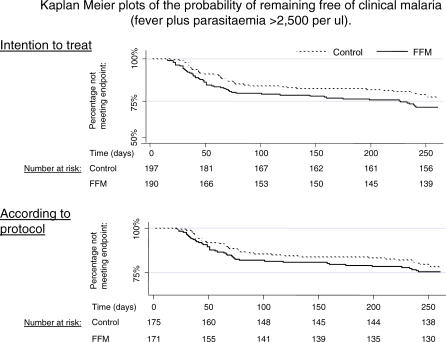
Primary Analysis of Efficacy The probability of remaining free of clinical malaria is plotted over the 9 mo of monitoring (the primary analysis). Numbers of children at risk are given below the Kaplan-Meier plots for ITT (top; *p* = 0.55) and ATP (bottom; *p* = 0.14). Both plots use an endpoint of over 2,500 parasites/μl and fever.

The time to first episode was shorter in the malaria vaccine group (Figure 2). The cumulative incidence of malaria (fever with parasitaemia over 2,500/μl) in the ITT group was 52/190 children (27%) in the malaria vaccine group and 40/197 in the control group (20%), (stratified log-rank test χ^2^ = 2.2, *p* = 0.14 for log-rank), with a hazard ratio of 1.5 (95% CI 1.0–2.3). ATP vaccinations were given to 346 children and followed up. Of these, 40/175 (23%) had malaria in the malaria vaccine group compared to 36/171 (21%) in the control group (stratified log-rank test χ^2^ = 0.36; *p* = 0.55; hazard ratio 1.3; 95% CI 0.83–2.1).

#### Ancillary analyses.

In a secondary analysis, as specified in the Report and Analysis Plan (Text S1), episodes were defined as fever with parasitaemia at any density. In the malaria vaccine group 69/190 children (36%) had at least one episode, and 54/197 (27%) in the controls (stratified log-rank test χ^2^ = 2.79; *p* = 0.095; hazard ratio 1.5 [95% CI 1.0–2.1]), and among those vaccinated according to protocol, 58/175 (33%) and 48/171 (28%), respectively (log-rank test χ^2^ = 1.26, *p* = 0.26).

**Figure 3:**
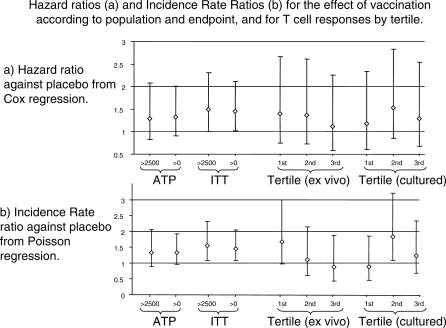
Secondary Analyses for Efficacy The results from secondary analyses of malaria episodes are shown. These multiple analyses were generated by varying the case definition and the statistical methodology, and are shown for both ATP and ITT. The more rigorous case definition (over 2,500 parasites/μl and fever) is the first in each group of comparisons. Cox regression was used to estimate hazard ratios for time to first episode (A) and Poisson regression was used to estimate incidence rate ratios for the frequency of episodes (B). Models were adjusted for age, village, and ITN use. The two groups of three points to the right of each panel show the hazard ratios and incidence rate ratios for subgroups FFM ME-TRAP-vaccinated children. Participants were divided into tertiles based on either ex vivo or cultured ELISPOT responses. Hazard ratios and incidence rate ratios for each tertile relative to the control group are shown, using a case definition of parasitaemia over 2,500/μl and fever.

Multiple episodes were modelled by Poisson regression, adjusted for the same covariates (Figure 3). There were 0.46 episodes of malaria (fever with parasitaemia over 2500/μl) per person-year among the FFM ME-TRAP vaccinees, and 0.36 episodes per person-year among the control group (incident rate ratio 1.6 [95% CI 1.1–2.3], *p* = 0.017). Among those vaccinated according to protocol, the incidence rate ratio was 1.4 (95% CI 0.89–2.1; *p* = 0.16). When these analyses were repeated using malaria definition of fever with any parasitaemia, the incidence rate ratios were 1.5 (95% CI 1.1–2.0), *p* = 0.013 for ITT and 1.4 (95% CI 0.96–1.9), *p* = 0.08 for ATP.

Only two episodes of malaria required hospital admission (one in each vaccination group), and neither of these met accepted criteria for severe malaria.

#### Secondary analysis by subgroup.

The Report and Analysis Plan had considered the possibility that susceptibility might vary by T cell response to vaccination (either by ex vivo or cultured ELISPOT). Vaccinees were divided into tertiles according to the ex vivo or cultured ELISPOT responses measured one week after vaccination, and each tertile was compared with the control group (Figure 3). No clear trend for differential susceptibility according to immunogenicity was seen, and a log likelihood ratio test suggested that the two models using immunogenicity (ex vivo and cultured) were not significantly better than using covariates alone (log likelihood testing gave *p* = 0.4 for ex vivo and *p* = 0.24 for cultured). There was no indication that immune response predicted susceptibility when multiple episodes were analyzed (*p* = 0.4, *p* = 0.29, respectively, for log likelihood).

However, the arithmetic mean was only 228 spots per million on ex vivo ELISPOT in the highest tertile. Exploratory analysis (not specified in the Report and Analysis Plan) divided vaccinees into seven groups of 28 children per group (on average), according to ex vivo T cell response. The highest responders were at a mean of 325 spots per million, but still had more frequent episodes of malaria than the control group (hazard ratio 2.39 [95% CI 1.1–5.9]). The 27 highest responders by cultured ELISPOT were at mean response of 1,130 spots per million, and the hazard ratio was 1.76 (95% CI 0.78–4.0) compared with controls.

Other endpoints were haemoglobin and parasitaemia detected at cross-sectional surveys at 3 and 9 mo. These variables were not different between vaccination groups. The prevalences of parasitaemia for FFM ME-TRAP and the control group were, respectively, 29% and 26% at 3 mo (*n* = 346) and 33% and 33% at 9 mo (*n* = 306). Mean haemoglobin was 11 g/dl (95% CI 10.8–11.1) before monitoring began (*n* = 362), and 10.1 at 3 mo (95% CI 9.8–10.5) in both the FFM ME-TRAP and control group (*n* = 354).

#### Immunogenicity.

**Figure 4:**
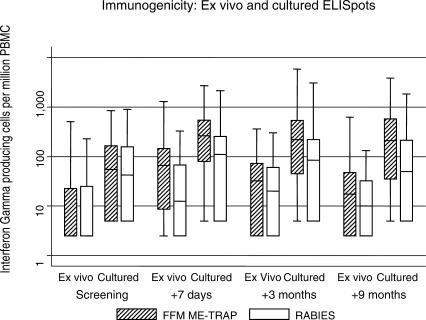
Immunogenicity T cell responses to vaccination identified by both ex vivo and cultured ELISPOT are displayed over time. Median, 25th, and 75th quartile, 5th and 95th quartile, and outlying results are given by box and whisker plots. Data were available for 400 children at screening (i.e., prevaccination), for 379 children 7 d after the last vaccination, for 345 at 3 mo, and for 304 at 9 mo. T cell numbers were similar at baseline for ex vivo (*p* = 0.4) and cultured (*p* = 0.91) responses. Ex vivo responses were higher among FFM vaccinees at 1 wk (*p* < 0.001) and 9 mo (*p* = 0.015), as were cultured responses. Ex vivo responses did not significantly differ at 3 mo (*p* = 0.27), but cultured responses did (*p* < 0.001).

The vaccine was moderately immunogenic (Figure 4). T cell responses were measured using both the ex vivo and the cultured ELISPOT, and both assays detected a response to vaccination. This response was significantly greater than that measured prevaccination (*p* < 0.001), and greater in FFM ME-TRAP vaccinees than in children receiving control vaccinations (*p* < 0.001). Ex vivo T cell responses rose from 30 spots per million before vaccination (95% CI 21–40) to 107 spots per million (95% CI 88–127) after vaccination. Cultured responses rose from 123 spots at baseline (95% CI 104–142) to 407 (95% CI 349–464). Vaccine-induced responses for both ex vivo and cultured ELISPOT remained above the control group at 9 mo (the final time point) (*p* = 0.015 for ex vivo, *p* < 0.001 for cultured ELISPOT).

### Adverse Events

The vaccine was well tolerated. Ten serious adverse events occurred. Four occurred after rabies vaccination (severe malaria, gastroenteritis, trauma, and asthma) and six after FFM ME-TRAP (severe malaria, an abdominal skin infection distant to the vaccination site, trauma, gastroenteritis, and multiple seizures). The serious adverse events identified were not unexpected events given the study population, were not closely related to vaccination in timing, and were judged unlikely to be linked to vaccination by the investigators and the Data Safety Monitoring Board. Local cutaneous discolouration and blistering was frequent, but blistering with a diameter greater than 0.5 cm occurred at a rate of 1%–4% (Table 2). No blisters were greater than 1 cm in diameter or associated with reports of marked pain. Keloid formation or hypertrophic scars were not seen. The ranges of routine haematology and biochemistry results, and frequency of abnormal results, were similar for the two groups. No abnormal laboratory results were attributed to vaccination.

**Table 2:**
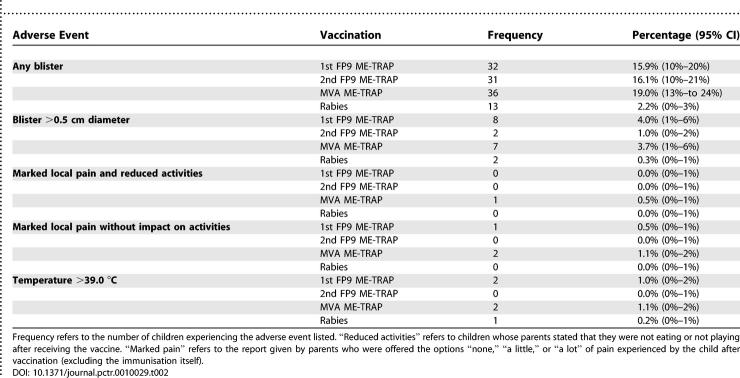
Solicited Adverse Events: Frequencies of Children Experiencing the Named Adverse Events at Any Time during the First Week of Monitoring

## DISCUSSION

### Interpretation

FFM ME-TRAP was safe when given to 1- to 6-year-old children in a malaria-endemic area. It was moderately immunogenic, but not as immunogenic as in malaria-naïve [8] or semi-immune adults [26], and it was not protective against episodes of clinical malaria. Although more malaria episodes were observed in the malaria vaccine group, the difference was not significant by primary analysis (ITT).

Although the formal possibility exists that altered peptide ligand effects [27], transforming growth factor β production [28] and T cell anti-inflammatory responses [29] induced by vaccination might increase susceptibility to malaria, there was no indication in our study that higher T cell responses predicted greater susceptibility to malaria within the vaccination group (either cultured or ex vivo). Since naturally acquired responses were not associated with protection [30], it seems unlikely that vaccine-induced responses could lead to greater susceptibility by suppressing naturally acquired responses. The DNA-MVA regime tested in Gambia induced stronger T cell responses to TRAP than seen here, without evidence of enhanced susceptibility [10]. There is therefore no obvious hypothesis to link vaccine-induced responses with enhanced susceptibility, and the significance testing by primary analysis indicates that this was a chance finding.

### Overall Evidence

FFM ME-TRAP was only moderately immunogenic in this population, despite the strong immunogenicity observed previously. Stronger immunogenicity was seen in previous Phase 1 studies in adults and children in Kilifi. Arithmetic mean responses by ex vivo ELISPOT were 610 spots per million PBMCs in malaria-naïve adults [8] but 350 spots per million in semi-immune adults in Gambia and 360 spots per million in Kenya [19]. The arithmetic mean T cell response during Phase 1 trials in children in Vipingo, Kilifi (where the EIR is 1 [23]) was 200 spots per million. In the Phase 2b study in Junju reported here (where the EIR is 22–53 [23]), arithmetic mean responses were 110 spots per million. Immunogenicity was moderately reduced for semi-immune adults compared with malaria naïve adults, and was reduced further among children in a malaria-endemic area. Several studies have shown dendritic cell function to be impaired by malaria and other chronic infections [31], and placental malaria induces T cell regulatory responses at birth [32]. There may therefore be a causal association between reduced immunogenicity and more frequent malaria and other chronic infections.

Different batches of FP9 ME-TRAP can be associated with different immunogenicity [19], and work to assess factors that may underlie this variability is in progress. However, studies on semi-immune and naïve adults used the same batch of vaccine, and preliminary studies in the same group of semi-immune adults suggested that the batches used for Phase 1 and Phase 2b trials in children had similar immunogenicity. It is therefore unlikely that most of the observed variation in immunogenicity is accounted for by batch variation.

### Generalisability

Active case detection might produce a less specific endpoint by detecting milder and more self-limiting illness than would passive case detection. This problem can be countered by using a parasitaemia threshold to define malaria cases [22]. It is conventional to perform blood film examination on history of fever alone, since relying on a single measurement of fever can miss cases [33]. However, subjective impressions of fever are extremely nonspecific [34]. In order to improve the specificity of cases identified by active case detection, we did not perform blood films from children with a history of a fever without an objective fever, but after the initial visit returned on three occasions in the next 24 hours to measure the temperature again. This approach appeared to be safe in this trial.

Is it nevertheless possible that a case definition with poor specificity obscured vaccine efficacy? This seems unlikely. Febrile malaria was identified in only five (6%) of 74 5- to 6-year-olds, compared with 19 (29%) of 65 1- to 2-year-olds, although fever and asymptomatic parasitaemia were common in both age groups. A study using passive case detection would probably require a 4-fold increase in sample size for similar power [35].

It is unclear whether reductions in liver parasites similar to those seen in sporozoite challenge [9] might have occurred in this trial without a reduction in clinical episodes, but these reductions might be expected among children with the highest T cell responses. These data, together with the Phase 2a and 2b trial data for RTS,S/AS02 [35,36] suggest that greater than 90% reductions in liver parasites are required for protection in the field. This trial also suggests that similar pre-erythrocytic vaccines should achieve T cell responses considerably higher than those seen here in order to provide measurable efficacy in children, and raises the possibility that T cell-inducing vaccines may show lower immunogenicity in higher-transmission settings. Further development of potent T cell-inducing vaccinations against malaria will examine different vector combinations and antigen inserts, in order to improve both immunogenicity and the breadth of responses induced.

## SUPPORTING INFORMATION

CONSORT Checklist Click here for additional data file. (48 KB DOC)

Trial Protocol Click here for additional data file. (135 KB PDF)

Alternative Language Abstract S1 Translation of the Abstract into Kiswahili (23 KB DOC)

Text S1 Report and Analysis Plan (37 KB DOC)

## Abbreviations

ATP: according to protocol
CI: confidence interval
EIR: entomological inoculation rate
ELISPOT: enzyme-linked immunosorbent spot
ITN: insecticide-treated net
ITT: intention to treat
MVA: modified vaccinia virus Ankara
PBMC: peripheral blood mononuclear cell
