# Supplementary material for: A Phase 2b Randomised Trial of the Candidate Malaria Vaccines FP9 ME-TRAP and MVA ME-TRAP among Children in Kenya
Source: PLoS Clin Trials. 2006 Oct 20;1(6):e29. doi: 10.1371/journal.pctr.0010029 (PMC1617125; doi:10.1371/journal.pctr.0010029)
Supplement: Trial Protocol [file pctr.0010029.sd002.pdf]

1) Title of project

Safety, immunogenicity and efficacy against febrile malaria of the candidate vaccines FP9 ME-TRAP and MVA ME-TRAP for children in an endemic area. (Version 2, 21/10/2004).

2) Investigators

Dr Philip Bejon (PI)
Dr Tabitha Mwangi
Mr Oscar Kai
Ms Jedidah Mwacharo
Dr Sassy Molyneux
Mr Brett Lowe
Dr Norbert Peshu
Professor Kevin Marsh
(Kenya Medical Research Institute, Centre for Geographic Medicine Research – Coast)

Dr Trudie Lang
Paul Milligan
Professor Adrian Hill
(Nuffield Dept. of Clinical Medicine, University of Oxford)

3) Abstract

About 1 million people die each year from malaria and there is no vaccine near to licensure. The Oxford group uses a heterologous prime-boost strategy, using sequential immunisation with different vectors delivering a common antigen (ME-TRAP: multiple pre-erythrocytic stage epitopes and the whole pre-erythrocytic stage antigen TRAP). Malaria challenge studies with sporozoites from *P. falciparum*-infected mosquitoes have been used to evaluate these vaccines: some individuals have been fully protected and many immunized groups show mean delays in time to parasitaemia of about 2 days corresponding to a killing of about 90% of malaria parasites in the liver. We have found these vaccines to be safe and immunogenic among adults and children in Kenya.
This study will evaluate the efficacy of the regime of FP9:ME-TRAP (attenuated Fowlpox virus recombinant for ME-TRAP) followed by MVA:ME-TRAP (modified Vaccinia Ankara recombinant for ME-TRAP) in 1-6 year old children in Kilifi District, Kenya, during two 3-4 month surveillance periods spanning the malaria transmission seasons. The trial will be randomised and double blind, using rabies vaccine as control. We will screen 450 children, aiming to recruit 410 children to be randomised in a 1:1 ratio to active or control vaccinations. Safety and immunogenicity data will be evaluated by clinical assessments and blood tests. Subsequent weekly follow up will allow blood film examinations for all febrile children. Rates of development of febrile malaria and proportions of children with episodes of febrile malaria will be compared between groups to determine efficacy.

4) Introduction (see appendix for abbreviations used)

Background

Research on T cell mediated protection against malaria has been in progress for several years in the Gambia and Kilifi, Kenya, supported by Oxford University, the Wellcome Trust and others. This has progressed from studies on naturally acquired immunity to experimental vaccination; Phase 1 trials in Oxford, the Gambia and Kilifi. We now propose an immunogenicity and efficacy trial.

Sporozoite immunization
Multiple immunization of human volunteers with irradiated sporozoite induces sterilizing immunity. This does not correlate with antibody titres against sporozoites, and is not generated by sporozoites made incapable of invading hepatocytes by over-irradiation [1]. In mice, T cell clones from irradiated sporozoite immunized mice transfer protective immunity to live sporozoite challenge [2]. These clones recognized circumsporozoite protein derived peptides on infected hepatocytes leading to cell lysis and parasite death [3]. Furthermore, gene knock out mouse experiments demonstrated that protection is dependent on interferon gamma production [4].

T cells in natural exposure.
The HLA-B*53 allele was associated with protection against severe malaria in a large case-control study of Gambian children, suggesting a role for CD8 positive T cells [5]. It was subsequently confirmed that T cells are induced by natural infection: Adults in endemic areas develop HLA-B*53 restricted T cells recognising a conserved nonamer peptide from liver-stage antigen-1 [6], and a peptide-based approach using allele-specific motifs identified fourteen other class I epitopes in six pre-erythrocytic P. falciparum antigens [7]. Such T cells exist in two forms: immediate effector cells, capable of responding in vitro after overnight stimulation by antigen, and a memory population, requiring 10 days stimulation with antigen and interleukin 2. These two populations do not correlate with each other [8] in individuals naturally exposed to malaria. Further, although the numbers of immediate effector T cells responsive to pre-erythrocytic antigen did not correlate with protection against mild malaria in children in Kilifi [9], cultured memory cells correlate with protection against natural infection in semi-immune adults given either RTS,S or a placebo vaccination [10] and possibly with protection from experimental infection of non-immune volunteers immunised with the RTS,S vaccine [11]. Both cell populations appear to correlate with protection after prime-boost vaccination.

Animal studies in Oxford
Sporozoite immunisation is expensive and impractical. In Oxford, BALB/c or C57BL/6 mice were challenged with *P. berghei* malaria after immunisation with recombinant vectors. The antigen TRAP was chosen for immunisation as a well-characterised pre-erythrocytic antigen with a homologue that is protective in rodent models and with less polymorphism than the circumsporozoite protein (CS) [12]. Various potential vaccine types delivering this antigen were compared; recombinant particles, peptides, plasmid DNA, numerous adjuvants, recombinant BCG, Salmonella, adenovirus, modified vaccinia virus Ankara (MVA) and attenuated fowlpox (FP9) . Most of these approaches induced only modest levels of T cell immunogenicity that invariably failed to protect against sporozoite challenge. However, a priming immunisation with plasmid DNA encoding an entire *P. berghei* pre-erythrocytic antigen followed by a booster immunisation with a recombinant modified Vaccinia virus

Ankara (MVA) vector carrying the same antigen induced complete protection in mice otherwise highly susceptible to sporozoite challenge[13]. Protection was associated with the number of peptide-specific gamma-interferon secreting CD8+ T cells measured in gamma-interferon ELISpot assays. Chimpanzee studies also show priming with DNA and boosting with MVA generates very high levels of T cells responding to TRAP[14]. This vaccination approach with heterologous immunisations; that is, two carriers encoding the same antigen, has become known as prime-boost. Attenuated fowlpox (FP9) can also be used as a priming agent. In mouse studies, sequential immunization with FP9 and recombinant modified vaccinia virus Anakara (MVA) recombinant for malaria antigen was more immunogenic and protective than DNA/MVA prime boost immunization [14]. Better protection was not achieved by sequential (triple) immunization with a DNA vaccine, FP9, and MVA.

Human volunteers

We used these recombinant vectors to vaccinate volunteers with a common antigen: ME-TRAP, comprising multiple pre-erythrocytic stage epitopes (ME) and the whole pre-erythrocytic stage antigen Thrombospondin Related Adhesion Protein (TRAP). Multiple DNA priming followed by intradermal MVA boosting induced 10-fold higher T cell responses than previously achieved with homologous immunisations. Partial protection against experimental malaria was manifest as a 2 day delay in time to parasitaemia after experimental malaria sporozoite challenge in Oxford [15]. The regime DNA:ME-TRAP followed by MVA:ME-TRAP boosting was then subjected to Phase I and II trials in the Gambia. 300 semi-immune adults were immunised and followed weekly for asymptomatic parasitaemia. The vaccination was safe, immunogenic, but not significantly protective (10%, 95% confidence interval -10 to 20%, Moorthy et al, PLOS Medicine 2004, In press.)

Since mice have greater protection with FP9 for priming instead of DNA, 17 volunteers were immunized with two fowlpox immunizations followed by MVA once. This was equally immunogenic, but more protective: 2 volunteers were completely protected after sporozoite challenge in Oxford, and the overall delay to parasitaemia was 2.5 days (p=0.32 for comparison with DNA/MVA combinations, p=0.0022 with unvaccinated controls). One of these two volunteers was protected on re-challenge at 6 months and 20 months. As in the mouse model, combining DNA and FP9 priming did not improve protection [16].

A recent analysis of PCR data allows calculation of the liver stage parasite numbers in volunteers after sporozoite challenge. This confirms the 90% reduction in parasite numbers predicted by the delay to parasitaemia[17].

Modified Vaccinia Ankara (MVA)

MVA is much safer than vaccinia because it fails to replicate in vivo. During the smallpox eradication campaign vaccinia scarification was associated with significant side effects. The most comprehensive data come from a 10 state survey of the USA in 1968: there were 2.8 cases of post-vaccinial encephalitis, 0.9 cases of progressive vaccinia, 10.4 cases of eczema vaccinatum, 23.4 cases of generalised vaccinia and 25.4 cases of accidental infection per million primary vaccinations with non-attenuated vaccinia[18]. In an attempt to reduce these side effects several attenuated strains of vaccinia were developed. The most widely used of these was MVA, derived by multiple serial passage of vaccinia strain Ankara through chick embryo fibroblasts. MVA caused no serious side effects when given by intramuscular, subcutaneous and intradermal routes to 120,000 individuals in southern Germany and Turkey [19]. MVA was safe when administered to neonatal mice by intracerebral and intraperitoneal routes, and did not disseminate in mice immunosuppressed by irradiation [20]. It appears to be attenuated as a consequence of genetic deletions, with a unique cytokine receptor profile which may explain it’s immunogenicity and safety.

The safety data acquired during 100 immunisations with prime boost regimes in healthy volunteers in Oxford, over 200 immunisations with MVA in the Gambia and 29 adults in Kilifi, Kenya has been favourable [16,21-23]. Local reactions occurred in 70% of subjects, limited to discolouration, blistering and itching, typically resolving over one week. 25% had a mild flu-like illness for not more than 24 hours after the first dose of MVA. These side effects are attenuated if MVA is given after FP9. Stocks of MVA encoding ME-TRAP are produced to GMP standard by Impfstoffwerk Dessau-Tornau, Roslau, Germany.

Attenuated Fowlpox (FP9)

Fowlpox is an avian poxvirus which causes disease in chickens but not mammals. Recombinant attenuated fowlpox has been used as a rabies vaccine in mammals. FP9 was derived from wild type fowlpox by 400 passages in tissue culture, leading to marked attenuation and loss of pathogenicity in chickens, including one-day old chicks[24,25]. In the UK a clone of FP9 has been developed and characterised in detail at the Institute for Animal Health in Compton[26]. The stock of FP9 from which FP9 ME-TRAP was manufactured was derived from a sample obtained of this clone. Like all avipoxviruses FP9 and FP9 ME-TRAP cannot replicate in mammalian cells. Attenuated strains of fowlpoxvirus and other avipoxvirus have been used safely as recombinant viral vaccines in many human clinical trials and have been demonstrated to be non-virulent in human volunteers[16,27-29]. 40 volunteers have been immunised on 1-2 occasions with FP9 ME-TRAP in Oxford and 16 with 2 immunisations in the Gambia. We have since immunised 29 adults in Kilifi. Safety data resembles that for MVA, limited to self limiting local reactions: discolouration and blistering lasting 1 week at maximum, and flu-like illness in 25%. As for MVA, GMP standard production is by Impfstoffwerk Dessau-Tornau, Roslau, Germany.

Previous Studies in Kilifi

We have immunised 29 adults in Kilifi with FP9 and MVA ME-TRAP combinations, and 24 adults with FP9 and MVA combinations encoding CSO. Immunogenicity has been marked and no safety concerns have emerged. (see appendix). Immunogenicity is slightly higher than that observed in Oxford and cross reactive to non-vaccine strains, and we detect high numbers of memory cells. 2 HIV positive adults have been immunized with a single, half dose of FP9, and 1 HIV positive adult with a half dose of MVA; more immunizations are planned. We are immunizing 18 children with FP9 prime followed by MVA boost. Preliminary data has shown minimal local and systemic reactogenicity, and we are currently engaged in the latter part of this study to optimize vaccine dose.

Studies of protection against asymptomatic parasitaemia in semi-immune adults

A previous field trial in Gambian semi-immune adults assessed two DNA and one MVA:ME-TRAP immunisations.and observed an efficacy of only 10% (P = NS) in reducing rates of asymptomatic parasitaemia. However, efficacy of FP9-MVA is likely to be higher than DNA-MVA vaccination and efficacy against disease in children should also be higher than efficacy against parasitaemia in adults. The reasons for this are described below.

- Early acquisition rates of parasite positivity are much lower than expected from calculations of entomological inoculation rates [44].
- Parasite incidence rates should be corrected for frequency of mosquito biting; evidence suggests this is proportional to surface area, and so lower for children than adults[45]. Reanalysis correcting for surface area would then suggest lower infection rates in adults.

- In both the field study of DNA:ME-TRAP prime, MVA ME-TRAP boost and the study of RTS,S conducted in the Gambia, gradually increasing protection against asymptomatic parasitaemia was seen when comparing older with younger age groups [46].
- Recent work suggests that blood stage immunity to a merozoite antigen protects against parasitization in a longitudinal study of adults after curative chemotherapy [47]. This is further evidence that incidence studies in semi-immune adults are complicated by naturally acquired immunity. This immunity would act after parasites emerge from the liver, but before they reach sufficient numbers to be seen by microscopy.

Furthermore, in endemic areas infection rates of adults are so high that it is very hard to observe efficacy against infection, in effect the experimental model is “saturated”. When studies following parasitaemia clearing chemotherapy have been conducted in areas at approximately ten fold different transmission rates (Northern Ghana [43] and Mali [48]), the rate of acquisition of parasitaemia during follow-up was very similar.

Finally, there is evidence that other measures reducing pre-erythrocytic parasites do reduce clinical disease despite a limited action on parasitaemia rates.

- Extensive clinical trials on bednets have been performed, and the data subjected to meta-analysis recently. Limited impact on parasitisation rates per se is demonstrated (10%), albeit studied in cross-sectional surveys rather than longitudinal cohort studies after radical cure. Efficacy is harder to demonstrate in areas at higher transmission rates. Nevertheless, 40-60% efficacy against mild malaria in young children is described, as well as efficacy against anaemia and severe disease in larger studies [49].
- During both the RTS,S study and the DNA/MVA:ME-TRAP study in the Gambia, although use of bed nets was not randomised, bed net use was not associated with any lower incidence of malaria parasitisation [46].
- Despite limited impact parasitisation incidence by the start of the rainy season (relative risk of 1.5) the risk of clinical disease changes dramatically (relative risk of 4.4)[42].

In summary the incidence rate of parasitaemia in semi-immune adults is a measure that appears to be insensitive to a ten fold reduction in malaria challenge rate, with between-individual variation determined heavily by blood stage immunity, and this incidence rate is poorly predictive of the potential protection afforded against disease in children, the main group requiring protection by a vaccine.

Importantly, a field trial in the Gambia demonstrated an overall 34% efficacy of RTS,S against asymptomatic parasitaemia in adults; 70% in the first 9 weeks, and none thereafter. However, phase I trials of RTS,S in non-endemic areas demonstrate longer lived efficacy (up to six months) against experimental challenge with a large inoculum of sporozoites [50]. The discrepancy between the duration of efficacy in these studies may reflect the limitations of studying time to asymptomatic parasitaemia in adults, and the protection seen against disease in children may be greater than that predicted by that seen for asymptomatic semi-immune adults.

Laboratory assays

When assessing immunogenicity in clinical studies we use the gamma-interferon enzyme-linked immunospot (ELISpot) assay in two forms. In its *ex vivo* form this assay correlated directly with protection in two mouse models of malaria [30]. In its short-term cultured form, it correlated with protection in the field trial of RTS,S/AS02 in the Gambia mentioned earlier. We assay with pools of 20-mer peptides spanning TRAP and all the epitopes for the ME string. The ELISpot enumerates T cells in volunteers' peripheral blood which secrete gamma-interferon on contact with an epitope from the ME-TRAP construct. Gamma-interferon secreted by T cells after interaction with infected liver cells has been shown to induce death of liver-stage parasites[31].

Potential subjects with HIV

The need for an HIV test before immunisation would preclude widespread delivery of a successful immunisation, and it is clearly desirable to protect HIV infected individuals as well as uninfected. These highly attenuated vectors are unable to replicate in human cells, and are very likely to be safe in HIV: this is confirmed by safety data for these vectors for adults in Germany [32] and New York [33]. We have immunised 3 adults with HIV infection in Kilifi, and plan to immunise at least 3 more over the next few months. (SSC proposal no. 759). We therefore propose to not include HIV testing as part of the health screen for prospective volunteers. This is likely to result in immunising 3-4 HIV positive children, and should not affect efficacy results.

Subjects with asymptomatic malaria parasitaemia or helminth infection at immunisation

Asymptomatic parasitaemia is common in the population eligible for preventative malaria vaccines. Previous studies have used antimalarials prior to each vaccine dose [34,35], because of concerns about immunogenicity. However, others have compared parasitaemic and aparasitaemic children, and suggested greater efficacy in the children with parasitaemia[36]. There is also data that T cell responses to immunisation with tetanus toxoid [37-39] and BCG [40] may be reduced by symptomless helminth infections. Certainly, it would be impractical to clear either parasitaemia or intestinal worm infections from children a prior to vaccination on a large scale, and an important component of this trial will be to examine whether parasitaemia or helminth infection at screening has an impact on immunogenicity.

Outcome measures

Such efficacy trials depend critically on the incidence of malaria in order to show differences. Symptomless parasitaemia and non-malarious fever are both common, and so frequently coincide by chance. This reduces the specificity of the trial endpoint, effectively reducing power (T Mwangi, Ph.D. thesis). For example, in a simulation over 12 weeks, where the incidence of malaria and non-malarial fever is 6% per week, vaccine efficacy 50%, and blood film sensitivity and specificity 90%, then the measured vaccine efficacy is 38%. Reducing specificity of the blood film to 80% reduces measured vaccine efficacy to 32%.

The use of parasite density thresholds in peripheral blood improves the specificity of diagnosing malaria. Although historically a cut-off of 5,000 parasites per ul has been used, the optimal threshold varies among populations, shown by calculating the malaria attributable fever fraction (ie the fraction of fever caused by malaria) for different populations. (T. Mwangi, Ph.D thesis). This method uses logistic regression with the outcome variable fever (present or absent) and explanatory variable parasite density. This model allows a theoretical risk of malaria to be given to each parasitaemic individual. Summing these risks in a population allows the malaria attributable fever fraction to be calculated.[41] We therefore propose to use this model to derive attributable fever fraction as

one outcome measure, and to derive a threshold parasitaemia to define febrile malaria episodes. The time to first episode will form the main outcome measure.

5) Justification

Justification
We believe a Phase IIb trial is warranted, to determine whether immunisation with two FP9:ME-TRAP primes followed by boosting with MVA:ME-TRAP is protective against clinical disease in 1-6 year old children, based on the significant efficacy data observed in the sporozoite challenge studies in the UK.

A previous field trial in Gambian semi-immune adults assessed two DNA and one MVA:ME-TRAP immunisations.and observed an efficacy of only 10% (P = NS) in reducing rates of asymptomatic parasitaemia. However, efficacy of FP9-MVA is likely to be higher than DNA-MVA vaccination and efficacy against disease in children should also be higher than efficacy against parasitaemia in adults. The reasons for this are summarised briefly.

Firstly, the protection seen in Oxford with FP9:ME-TRAP priming was greater, albeit non-significantly (p=0.3), than that seen with DNA:ME-TRAP priming. (p=0.3) This suggests a 70% probability that FP9:ME-TRAP priming is more protective than DNA priming in humans, as it is in mice [15,16].

Secondly, although it was originally suggested that incidence studies in semi-immune adults would be uncomplicated by significant naturally acquired immunity [42,43] this is now known not to be the case and there are strong reasons to believe that protection against asymptomatic parasitaemia in semi-immune adults would not be expected to accurately predict protection against clinical disease in children. the reasons for this are discussed in detail in the introduction. In summary, the incidence rate of parasitaemia in semi-immune adults is a measure that appears to be insensitive to a ten fold reduction in malaria challenge rate, with between-individual variation determined heavily by blood stage immunity, and this incidence rate is poorly predictive of the potential protection afforded against disease in children, the main group requiring protection by a vaccine.

This trial of these vectored vaccines is important despite the current status of development of RTS,S, which is now in phase IIb trials in children. RTS,S gives somewhat higher levels of protection in the challenge model than FP9-MVA (90% vs 95%) but its duration of efficacy in the field indicates that used alone it is unlikely to be adequate as a malaria vaccine. Culture ELISpot immunogenicity data and efficacy in 6 and 20 month post-vaccination challenges with FP9-MVA vaccination suggest that these vaccines may provide more durable protection. RTS,S is primarily an antibody inducing vaccine, directed against invading sporozoites. Combining RTS,S with a T cell inducing vaccine such as FP9-MVA targeting intra-hepatic parasites should offer greater protection than either alone. Demonstrating efficacy for each component alone in field trials will probably be necessary before combining immunisations.

6) Null Hypotheses

There is no significant difference between control and actively immunised children for the rate of development of or number of episodes of febrile malaria

7) Objectives

General Objectives

This study will evaluate the efficacy, safety and immunogenicity of the regime of FP9 ME-TRAP followed by MVA ME-TRAP in 1-6 year old children in Kilifi District, Kenya, in a randomised, double blind, placebo controlled trial.

Specific objectives

To measure the following in children immunised with a control immunisation (rabies vaccine) or FP9:ME-TRAP MVA:ME-TRAP;

- efficacy during two 3-4 month surveillance periods spanning the malaria transmission seasons. Rates of development of febrile malaria and proportions of children with episodes of febrile malaria will be compared between groups to determine efficacy.
- Safety data will be generated by comparing the incidence of solicited local side effects at the site of injection, and systemic side effects in the days following immunisation.
- Immunogenicity will be measured by studying the immediate effector T cell response and long term memory T cell response after vaccination. (see *Laboratory assays* under introduction). We will examine for a correlation with protection.

8) Design

Study site

The investigation will take place at the KEMRI Centre for Geographic Medicine Research – Coast, Kilifi. There are several dispensaries suitable for such a study in Kilifi district. We currently plan to use Gongoni dispensary, 30km south of KEMRI Centre for Geographic Medicine Research. Kilifi District experiences long rains in May-July and short rains in November/December. Gongoni is near a fresh water lake, and this may explain the continued presentation of malaria cases to Gongoni dispensary when other areas have little malaria. Measured Entomological Inoculation Rates in the area vary from 10-50 per year. The dispensary is the primary source of antimalarial treatment in the area: there are very few shops or private dispensaries in the area. The local administrative structure involves an area chief, a sublocation chief, a dispensary health committee and a clinical officer based in the dispensary. All these parties are keen to facilitate interventional research in the area. Adequate space exists in the dispensary to see children for clinic visits and immunisations.

Study population

There are 600 children between the ages of 1 and 6 years within a 2 kilometre radius of the dispensary. The local population are predominantly Mijikenda (Giriama), and subsistence farmers.

Sampling

A sample size calculation using a time to event, and assuming an even distribution of the risk of malaria, is based on a 60% risk of clinical disease during the first rainy season (estimate from previous studies in the area). To detect 40% efficacy with 90% power at p=0.05 requires 340 children in total, allowing for 30% probability of loss to follow up (spread over the period of monitoring) requires 372 children. To accommodate early loss to follow up, and incomplete immunisations, we aim to recruit 410 children, which we estimate will require 450 children to be screened for eligibility. Should the proportion of children with malaria attributable fever be only 40% because of poor rainfall, we would still have 90% power to detect 48% efficacy.

Sensitisation and recruitment

We would hold a series of public meetings to explain the study to the community. During this meeting the investigators will explain the following: the need for a vaccine including a simple picture of the burden of malaria on the community; the current status of vaccine development including the fact that this is likely to be a prolonged process; the study screening and informed consent procedure; risks of vaccination and the unproven benefits of vaccination. It will be stressed that this is an experimental vaccine, and cannot be guaranteed to provide protection and that it will therefore still be necessary to seek treatment for possible malaria even after vaccination. We will explain the need for a health screening before receiving vaccinations, since the vaccine is in an early stage of development.

After this meeting, we will ask potentially interested parents to register their names at the dispensary. A field worker will visit these parents at home. Should only one parent have attended the public meeting, this will allow both to be involved in discussions before consenting. Consent forms will be signed at this visit, and parents who feel that the trial is appropriate for their child after this meeting will be invited to attend a formal screening visit. This will include a further discussion of the study with the principal investigator, and an opportunity for further private discussion. It will be stressed that the children can be withdrawn from the study at any stage.

Outline of Procedures

Potential subjects, whose parents give informed consent, will be screened by history, medical examination and laboratory testing for exclusion criteria. Blood will be taken for immunology tests at baseline. Those eligible will be invited to return for 3 immunisations, spaced 4 weeks apart; FP9:ME-TRAP, FP9:ME-TRAP then MVA:ME-TRAP. Immunisations will be conducted in a local dispensary with facilities to treat anaphylactic reactions, with observation for 1 hour before leaving. These facilities will include all standard drugs, intravenous access equipment and airway management (including suction and oxygen). A doctor and nurse trained in paediatric resuscitation will be in attendance. Subjects will be visited to assess local and systemic reactions 1,2,3 and 7 days after each immunisation. A course of curative anti-malarials will be started one week after the third

immunisation. This will ensure that children developing fever during surveillance are less likely to have co-incidental chronic parasitaemia which would obscure the study endpoint. The study will be timed to complete this phase before the start of the malaria season. Surveillance for episodes of malaria will then begin. Children will be seen weekly, their temperature measured, and their parents asked about symptoms. Those afebrile but symptomatic will have their temperature measured again three times over the next 24 hours. Febrile children will have a blood film made, a rapid test for malaria parasites, and treated with anti-malarials if positive. Clinical disease based on febrile illness with blood film confirmed parasitaemia above a defined threshold will be the primary endpoint. The primary analysis will be the rate of acquisition of the first episode of clinical disease. Full details of these procedures are given below.

Role of the Local Dispensary

We plan to undertake all immunisations and follow up in the local dispensary. This will require installing oxygen delivery and suction (battery powered) in the local dispensary, as has been done for 2 other dispensaries in the district during phase 1 trials.
The single clinical officer working in the local dispensary will be supported in management of any children from the immunised cohort by the principle investigator, and also those outside the cohort should the clinical officer request it. We would undertake to provide co-artemether for use in the local clinic for children outside the cohort, to allow equitable access to healthcare. Since Government of Kenya policy is to supply this as first line treatment, this will be sustainable.

1. Screening visit.

We will take a clinical history, examine all children carefully and conduct a number of standard laboratory tests (see below) to screen subjects for clinically significant acute or chronic diseases. A height and weight will also be recorded. Bed net use will be recorded. A photograph of the child to aid identification on vaccination and assessment visits will be taken. Children will be given a serial number. Their three names, birth date, the names of their parents and their house location recorded. A photograph of the child and parent will be taken, and attached to the study record. We will assess bed net use during home visits for post immunisation follow up and surveillance.

Inclusion criteria
Children 1-6 years old who are in good health, and whose parents consent.

Exclusion criteria
Exclusion criteria will be:

- Clinically significant skin disorder, allergy, symptomatic immunodeficiency, cardiovascular disease, respiratory disease, endocrine disorder, liver disease, renal disease, gastrointestinal disease, neurological illness, severe malnutrition (mid upper arm circumference less than 11 cm).
- History of splenectomy
- Serum creatinine concentration above the age related normal range in Kilifi.
- Serum ALT concentration above the normal range in Kilifi

- Clinically significant anaemia, (ie. with symptoms of limited exercise capacity, or signs of a high cardiac output state; large volume pulse, heaving cardiac apex beat, resting tachycardia).
- Blood transfusion within one month of the beginning of the study
- History of vaccination with previous experimental malaria vaccines
- Administration of any other vaccine or immunoglobulin within two weeks before vaccination.
- Current participation in another clinical trial, or within 12 weeks of this study
- Any other finding which in the opinion of the investigators would increase the risk of an adverse outcome from participation in the trial.
- Likelihood of travel away from the study area

Urine will be collected for dipstick examination for blood and protein at screening only, to avoid immunising a child with sub-clinical glomerulonephritis. Stool will be collected for microscopy.

HIV testing
There is no requirement for host immunity to limit replication of the virus vectors, since they are only capable of a single round of replication *in vivo,* and no reason to suppose particular risk to HIV positive individuals. Indeed, we have now immunised 3 HIV positive adults in Kilifi, and safety data is reassuring for very similar vaccines in HIV positive adults elsewhere. We do not plan to include HIV testing in the screening tests before immunisation, and this is likely to result in 3 children with HIV being immunised. Should a severe adverse event be possibly vaccine related in the view of the Data Safety Monitoring Board, we would request parental consent for HIV testing of the affected child.

Blood sampling
5 mls of blood will be collected at screening, one week after final immunisation and 8 -18 weeks after final immunisation, and at the end of 9 months surveillance. The order of sampling at this latter timepoint will be assigned randomly. The following tests will be performed:

- Full blood count
- Serum ALT and creatinine
- *Ex vivo* ELISpot (to count IFN gamma producing T cells) and cultured ELISpot.
- Where possible, lymphocytes will be frozen for repetition of unusual results, or to explore specificity of responses with peptide pools, or after depletion of specific cell populations.
- Plasma stored to assay antibodies to TRAP or viral vectors.
- The pelleted red cell and neutrophils after PBMC isolation will be used for sickle cell and thalasaemia typing (electrophoresis and PCR typing respectively)
- Stool microscopy for helminths

Blood will be drawn into a paediatric size EDTA tube, and a glass lithium heparin tube. These tests will require 5 mls.

Parasitaemia at screening
Children with parasitaemia will not be excluded from study. It is unclear whether chronic parasitisation would be helpful or unhelpful in immunogenicity: other vaccine studies have

suggested that clearing parasitaemia might reduce the observed immunogenicity [36]. In the absence of clear evidence, we would not clear parasitaemia during the first two immunisations. Children with symptoms or signs at screening and parasitaemia will be treated. All children will be followed up closely during immunisation, including those with asymptomatic parasitaemia, and treatment given should they develop symptoms. Antimalarial treatment will be given to all children after the third immunisation to prevent chronic parasitaemia complicating the surveillance period. Similarly, children with signs or symptoms due to helminth infection confirmed by stool microscopy will be treated immediately. Those without will be treated after the immunisation is completed.

Anaemia at screening.
Children identified with anaemia (Haemoglobin less than 8.0 g/dl) will receive iron supplementation, and further standard investigation and management. If they have no clinically significant signs or symptoms on screening, they will still be eligible to enter the study.

Laboratory procedure.
Full blood counts will be performed on the EDTA anticoagulated blood. The lithium heparin tube will be centrifuged, and 1 ml plasma removed for biochemistry and storage. 1 ml RPMI will be added to the remaining blood, and PBMC separated by standard techniques. At screening, 1ml of the pelleted red cells and neutrophils will be taken for PCR and electrophoresis typing for thalasaemia and HbS respectively.

Plasma and cells will be stored at -20°C and -192°C respectively. *Ex vivo* and cultured ELISpot's are currently being performed in the immunology laboratory at the CGMRC, Kilifi, and results are validated by rigorous use of negative and positive controls. This technique uses either overnight or prolonged stimulation by antigen of separated lymphocytes from the child's blood sample to count the number of interferon gamma producing cells. Antibodies against MSP1-19, AMA and red cell variant markers will be measured.

Dissemination and explanation of blood results
All non immunology blood results will be given to and explained to all parents after screening and at follow-up visits. Immunology results will be explained in general terms, for the group not for individuals, at the end of the study. Those with abnormal blood results at screening will be offered appropriate investigations and treatment or referral as necessary. Parents will receive an explanation of all screening results before proceeding with immunisation. We will provide treatment should parents request treatment for symptomless malaria parasitaemia or intestinal helminth infections. Parents of children with symptomless malaria parasitaemia will be warned that they should present for assessment should they develop fever.

2. Vaccine administration

Randomisation
Children will be given serial numbers in the order of recruiting. A random allocation order in blocks will be generated by an independent statistician, part of the DSMB, and sent to the local safety monitor. The list of children will be ordered by age and sex, before being given to the local safety monitor. The local safety monitor will then apply the order of the

random block allocation to the children in the list given to him/her by the principle investigator.

A nurse will be employed to give vaccinations, separate from the investigators. Standard nursing training in Kenya includes considerable emphasis on correctly giving intradermal injections, because of the importance of the BCG programme, and a practical assessment is conducted in final exams. The nurses involved in this study will be required to show they have maintained the skill of giving these vaccinations recently. They will be briefed by the local safety monitor in the importance of maintaining blinding to the investigators. They will be shown the storage arrangements for vaccine, and the doses explained. A detailed SOP will cover this area. The investigator will inform the local safety monitor of the immunisations planned each day, who will instruct the nurses which immunisations these children are allocated to give. On the morning of each immunisation day, they will remove the required vaccines, placing them in a sealed box containing ice, for transport to the field.

Immunisation will be conducted in a designated room, and the investigators will not be present during immunisation. Nurses will document the precise details of immunisation (vaccine, number of injection sites, volume administered, vial and lot number) in folders separate to the main folder held by the investigators. They will only record that immunisations have been given in the main CRF. The vaccination records will be shown to the local safety monitor on return, to ensure no incorrect immunisations have been given, and stored in a locked cabinet not accessible by the investigators.

Storage and administration

The vaccines will be shipped from Oxford on dry ice then stored at -70^{0}C at the CGMR-C unit until required. Vaccines will be thawed on the morning of use, and kept in a cold box in the field. They will be used within 4 hours of thawing in the field. Should vaccines not be used during the day, they may be refrigerated overnight, but not re-frozen, and used the following morning.

Temperature checks and a systematic enquiry for symptoms will be made before each vaccination. Vaccination will be deferred in any child with a clinical illness (defined as signs of obvious clinical disease and/or a fever >37.5°C) on the day of vaccination. Medical treatment will be provided, including inpatient care if necessary.

Each child will be identified by name, parents name, age and photograph before vaccination. Vaccines will be administered intradermally in the left deltoid region with a fine gauge syringe and needle. For convenience, vaccine is drawn out of the vial with a 21 gage syringe and needle, before being transferred to the fine gauge syringe for injection. Children will receive oral vitamin A supplementation according to standard government guidelines (200,000 international units) when they attend for vaccination. This is advised for the expanded programme of immunisations, to ensure immunogenicity. Each child will be monitored for one hour (or longer if necessary) after each vaccination. Resuscitation (including intubation) equipment and medication will be available in the clinic site and a clinician trained in resuscitation present at all times. This will include installing facilities for delivering high flow oxygen and suction in the local dispensary.

The first and second immunisation will be with FP9:ME-TRAP, the third with MVA:ME-TRAP. These immunisations will be spaced about 28 days apart. Children who miss immunisation days will be immunised later provided the gap between immunisations is 35 days or less. We are currently completing a Phase 1 safety and immunogenicity study to determine the optimal dose. These data will be discussed with the DSMB before deciding between $5x10^{7}$ or $1x10^{8}$ for FP9:ME-TRAP and $7.5x10^{7}$ or $1.5x10^{8}$ for MVA:ME-TRAP.

Control

The Human Diploid Cell Vaccine for rabies will act as control. It can be given intradermally, requires three doses and will offer benefit in protecting against rabies. Rabies occurs in Kilifi district, pre-exposure vaccination reduces the likelihood of contracting the disease considerably, almost completely if combined with post-exposure vaccination.

This route is advised by the World Health Organization and by the Department of Health of the UK, using a 0.1ml dose. The standard course of immunisation is days 0,7 and 28, although days 0 and 28, days 0, 3, 7, 14 and 30 or days 0 and 3-7 are also advised depending on pre or post exposure use. We would use the vaccine on days 0, 21-28 and 42-60 to parallel the use of the candidate malaria vaccines. The same timing of vaccination has been used safely in 150 Gambian adults.

Procedure for management of intercurrent clinical malaria.

Any subject diagnosed with clinical malaria during the immunisation period will receive treatment according to national guidelines. If the child is well and afebrile by their next vaccination day, vaccination will continue according to the trial schedule.

Curative treatment for subclinical malaria

The presence of chronic parasitaemia during the follow up would complicate the assessment of vaccine efficacy. The first line antimalarial of choice according to Government of Kenya policy is co-artemether (a combination of artemether and lumefantrine). Given the relatively long half life of lumefantrine (3-6 days, with plasma levels becoming negligible by 35 days) this may complicate early assessment of vaccine efficacy. Artemether alone is highly efficacious, but requires daily therapy for one week [51,52]. Efficacy is then highly dependant on compliance, and for this reason the drug is not often used alone. However, during this study we will undertake directly observed therapy, beginning one week after immunisation, in order to ensure successful treatment. Malaria treatment at other times will not be directly observed (see below) and co-artemether will be used.

3. Immediate Assessment and follow up

A field worker will assess and record local adverse events; pain, itch, discolouration, blistering, ulceration and limited arm movement. He or she will then record systemic adverse events; temperature, headache, malaise and nausea. Each side effect will be graded 0 to 4;

Grade	Description	Example, using fever
0	Absent	
1	No impact on activities of daily living	Symptomless fever.
2	Activities of daily living can still be completed, but with difficulty	Feeding poorly due to fever.
3	Activities of daily living cannot be completed satisfactorily.	Missed meal because of fever.

The activities of daily living used to classify side effects if present will be eating, sleep and play.

This grading system does not classify serious adverse events. A side effect is serious if it causes hospital admission, death, incapacity or is life threatening. Discussion with the local safety monitor and external DSMB will determine if the event is suspected to be vaccine related, and whether the trial should be halted. The local safety monitor and DSMB will also decide if individual children having severe or serious side effects should or should not continue to receive further immunisations. Children receiving incomplete immunisations would still be included in subsequent surveillance and safety assessment.

Each child will be seen at clinic or visited at home on day 1, 2, 3 and 7 after each vaccination and if necessary the child will continue to be seen daily until the symptom(s) have resolved. In addition, on day 7 after the final vaccination blood will be taken for safety and immunogenicity tests (see Blood sampling above). On day 1 after the first immunisation, a record of bed net use will be made. The field worker will ask to observe the net, noting if it was positioned where the child slept, and will ask if the net has been treated. Standard Operating Procedures, Case Report Forms and practice will be reviewed by an independent safety monitor. Parents will be told of any safety concerns or abnormal blood test results. Summaries of adverse event profiles will be discussed with the local safety monitor and DSMB.

Day 7/Week 12 and end of study follow up

7 days after each immunisation, 12 weeks after the last immunisation and at the end of the study (9 months), we will ask parents to return with their children to the dispensary. They will be reviewed by the investigator, and assessments made for local and systemic toxicity as described above.

In addition, on 7 days after the last vaccine (peak immediate immunogenicity), 12 weeks after the last vaccine and at the end of the study, a blood sample will be drawn for an assessment of safety and immunogenicity as follows;

- Full blood count
- Serum ALT and creatinine
- *Ex vivo* ELISpot (to count IFN gamma producing T cells) and cultured ELISpot.
- Where possible, lymphocytes will be frozen for repetition of unusual results, or to explore specificity of responses with peptide pools, or after depletion of specific cell populations.
- Plasma stored to assay antibodies to TRAP or viral vectors.
- Blood film for parasitaemia (last time point only)

4. Surveillance

Children will be seen once weekly by a fieldworker. An axilliary temperature reading will be taken, and their parents will be asked for any symptoms of fever. If the child has a temperature, a finger-prick sample will be used to prepare a slide, and a capillary tube used to collect blood for a rapid malaria test (10 microlitres) and stored for PCR (a few microlitres onto filter paper). If the child has no temperature on measurement, but symptoms are reported, the fieldworker will return three times over the following 24 hours to repeat the temperature reading. An unplanned assessment can be requested by the parents on any day. On the rare occasion that children are found to be critically unwell, transport by landrover

back to KEMRI will be arranged. This will include other children not necessarily part of the study. The field workers will be able to make these arrangements by mobile telephone.

After 3 months of surveillance, a further record of bed net use will be made (as described above in the immediate assessment and follow up section).

Monitoring will continue for 8 months (mid May to mid January), to include both long and short rains. We consider it important to monitor for this length, since the long lived memory cells may be more important in protection than the shortlived effector response, and natural exposure during the first season may boost this response. At the completion of monitoring in mid-January there will be a final safety review of each child and blood sample taken for safety bloods and T cell responses.

Rapid malaria test

We have used the Unigold rapid diagnostic test for the falciparum HRP2 protein. Sensitivity and specificity of 99% are reported in the literature, and our experience confirms this. The false positive results appear to relate to persistence of antigen after parasite clearance, which should be infrequent in our cohort since follow up begins 4 weeks after curative treatment. Field workers will be trained in performing these rapid tests and gain experience in KDH outpatients before surveillance begins. The kits are designed to be performed and read by those without laboratory training.

Antimalarial treatment

We will dispense the first line antimalarial advised by the government of Kenya, co-artemether, labelled with the child’s name and other identifiers in advance of surveillance, together with instructions for use to be read to the parents by the field worker. The field workers will have mobile telephones, to allow discussion with the principal investigator before drugs are dispensed. It will be made clear to the parents that a blood test for malaria only has been performed, and that the child has not been seen by a doctor. A record will be made that this information has been repeated to the parent on each occasion that drugs are dispensed. Should the principal investigator feel a doctor’s assessment is required after discussion with the field workers, the parents will be asked to bring the child to the dispensary, where they will be met for assessment by a medically qualified member of the team.

We will work in close communication with the clinical officer based in the dispensary, and one field worker will be in contact each day to be informed should any of the study subjects present for treatment, so as to organise blood films and other assessments should they be required, according to the protocol for surveillance visits.

Blood films

Thick and thin films will be made in the field. These will be transported back for giemsa staining once air-dried. 100 high powered fields will be read in duplicate. Where greater than 30% disagreement over parasite numbers occurs, they will be read in triplicate. Results will be recorded on paper in the laboratory. This information will be entered into the CRF once available, along with a record of treatment.

Cross sectional study

All children in the study will have blood tests for safety (full blood count, ALT and creatinine and immunogenicity 3 months into surveillance and at the end of surveillance. A blood film examination will be included at the last blood test. Children with a haemoglobin lower than 8g/dl will be treated with iron supplements, and repeat haemoglobin

measurements made after 8 weeks of treatment. Further investigation will be indicated if the haemoglobin has not recovered.

Intercurrent illness.

The investigators will be responsible for treating intercurrent illness. Parents will be able to request reviews when visited by the field worker, who will inform the investigators. Children will be seen either in Kilifi District Hospital, or the local dispensary. In the extremely unlikely event that a child requires admission for a suspected vaccine related side effect, the local safety monitor will be asked to assume responsibility for the child's care, through other medically qualified persons. We would wish to avoid antibiotics with anti-malarial properties during the surveillance period, provided this is consistent with optimal clinical care. This primarily concerns co-trimoxazole, which has high resistance rates for most bacterial infections in Kilifi, and erythromycin, which is rarely used. Should either be required for best clinical care (eg. suspected pneumocystis carinii pneumonia) they will be used, but also should their use be at all considered in any other situation, the local safety monitor will be consulted.

Sponsor

Oxford University will take the role of the Sponsor, in taking responsibility for the trial, providing the clinical trial monitor(s), and being responsible for the persons directly managing the trial.

Data Safety Monitoring Board (DSMB)

A Data Safety Monitoring Board consisting of four persons will be set up to review and comment on both the clinical protocol and the reporting and analysis plan (RAP) before it can be implemented. The DSMB will be able to end the trial at any stage.

The main role of the DSMB is to review and advise on management of serious adverse events which occur during the trial. Furthermore after the end of the trial, the DSMB will be provided with a summary of the safety and reactogenicity data from the trial. As we expect decreased reactogenicity after the third dose compared to the first and second doses of the vaccination regime (based on previous Phase 1 studies), and no further interventions are planned during the surveillance period, no interim report will be prepared.

The DSMB may, if it deems it necessary, convene a meeting with or request further information from the Principal Investigators or The Local Safety monitor.

Local Safety Monitor

The overall role of the Local Safety Monitor, who will be based in Kilifi, will be to support the clinical investigators and to act as a link between the investigators and the DSMB. This would allow a safety monitor to examine a child with a potential serious adverse event, to confirm findings reported by the investigators. All serious adverse events will be reported to him or her. He or she will have the authority to suspend the whole or any specific aspect of the trial pending discussion with the DSMB. He or she will keep code break envelopes to unblind individual study subjects if deemed necessary for medical and/or ethical reasons.

Since most staff will not stay in Kilifi for the entire duration of the study, the role may be taken on by more than one person. This will be arranged prospectively.
The Local Safety Monitor's role will include advising the investigators on whether particular circumstances in a study warrants formal notification to the DSMB, and clinical advice on any illness in study subjects especially in circumstances in which treatment might influence the course of the trial.

Independent Clinical Trial Monitors

There will be one or more independent ICH-GCP clinical trial monitors who will monitor the trial. They will advise the investigators of areas where the trial is not compliant with ICH-GCP, and report to the sponsor.

Insurance

All staff are insured by insurance policies held by their employers: the University of Oxford or KEMRI.

9) Data Management

Data storage

Adverse events will be documented in individual case report files for each child. They will be recorded under two headings, local and general. There will be separate sections for concomitant medication, concomitant vaccination, non-serious adverse event documentation, serious adverse event documentation and study conclusion. Any deviations from the study protocol will be documented. Case report files will be kept securely.

Data will be entered in the CRFs in one of 3 ways. Data from clinic visits including screening, day 7 post vaccination follow up and medical assessments for fever will be entered directly in the CRFs carried to the dispensary on that day. Data acquired during home visits from days 1-3 post vaccination or surveillance for malaria will be entered on record sheets carried by fieldworkers. These will be transcribed to the CRFs on return from the field. Data from blood films will be transcribed to the CRF by the investigator.

Data management and analysis

Data entered into CRFs will be entered into a Visual Fox Pro™ data base as it is acquired. CRFs will be given to data entry personnel on return from the field after clinic visits. The forms generated by laboratory results and field trips will be supplied to them, after data is transcribed to the CRF by field workers or the investigators. This data base will contain the screening, post vaccination visits and surveillance, but not which vaccine was given. At the completion of the trial the code will be broken for analysis.

Analyses will be performed on subjects with intent to treat (ITT) and according to protocol (ATP).

Intent-to-treat cohort (ITT)

The intent-to-treat cohort will include all subjects enrolled in the study, who received at least one dose of the candidate malaria vaccines or comparator and for whom data for the observation in question are available.

Protocol-defined cohort for analysis of efficacy (ATP)
The protocol-defined cohort for analysis of efficacy will include all evaluable subjects (i.e., those meeting all eligibility criteria, complying with the procedures defined in the protocol, for whom data concerning efficacy endpoint measures are available.

Safety data.
All reactogenicity analyses will be on an intent to treat basis, comparing control to malaria vaccine. ALT, plasma creatinine, haemoglobin, white cell count, platelet values will be described by time and vaccine group.

Immunogenicity
Both ITT and ATP cohorts will be analysed, using both immediate *ex vivo* and cultured ELISpots. Immunogenicity will be examined by age, sex, presence of stool helminth infection and parasitaemia at screening. Geometric means of T cell numbers with 95% confidence intervals for central tendancy will describe groups.

Demographic data.
Age, sex and bed net use will be presented by treatment group to ensure comparability.

Efficacy
Efficacy will be analysed by both ITT and ATP.

Primary Analysis
Cox regression model analysis of incidence of clinical disease will allow assessment of the time to first episode as the primary outcome. Clinical disease will be defined by fever and parasitaemia above a threshold defined from logistic modelling of the risk of fever for parasite densities. Age, bed net use, blood stage immunity, sickle cell status and thalasaemia typing will be used as covarietes. It will also allow correlation of immune responses with protective efficacy.

Secondary analyses

- The total number of clinical disease episodes during surveillance, using age, bed net use and blood stage immunity as covarietes.
- the number of febrile malaria episodes in vaccine versus control groups, as calculated by the Malaria Attributable Fever Fraction, corrected by number of children and time of observation. This method does not assign a definitive status to each child, but produces a figure for the fraction of the group estimated to have febrile malaria. The cross-sectional bleed will be used to fit the model by providing the afebrile population, but will not be used in determining the Malaria Attributable Fever Fraction. The first half and second half of monitoring will be analysed separately.
- the other secondary analyses will compare mean haemoglobin concentrations during cross-sectional surveys, and the number of high parasitaemia (>500,000 per microlitre) infections in each group. The incidence of severe malaria will not be frequent enough to allow analysis, but data will be presented.

10) Time Frame

Pilot Study

Not applicable

Definitive study

Community sensitisation/recruiting	Jan 2005
Screening	1 Feb- March 2005
1st immunisations	March 7th-March 18th
Catch up immunisations	March 21th-24th
2nd immunisations	April 4-April 15th 2005
Catch up immunisations	April 18th-April 22nd
3rd immunisations	May 2nd-May 13th. 2005
Catch up immunisations	May 16-20th
Surveillance	May 16th – January 30th 2006

11) Ethical Considerations

The protocol will be submitted to both the KEMRI National Ethics Committee, and the Oxford Research Ethics Committee. Changes to the protocol requested by either committee will be referred to the other for approval. The Ethics Committees must be informed of serious and/or unexpected adverse experiences occurring during the study, and any new information that questions the safety of the subjects or the conduct of the study.

Human subjects

Confidentiality

All notes will be held securely in a filing cabinet in a locked office. Electronic databases will identify subjects by serial number only. For safe keeping, a second database linking serial numbers to personal information will be created at the end of the trial, and stored separately. This will ensure that rapid access to data should further follow up be required, and will allow passive monitoring of adverse events presenting to Kilifi District Hospital to continue after the end of the trial.

Duty to minimise risks to children

Adverse events are possible with any new vaccine. The risk is minimised by use of two candidate vectors chosen specifically for their good safety profile and their inability to replicate in humans. The comprehensive quality control, toxicology and manufacturing data to Good Manufacturing Practice and the absence of serious adverse events in phase I studies

in Oxford and the Gambia, and phase 1 studies for adults and children in Kenya, provides firm evidence of the minimal nature of risk in this study. However anaphylaxis is estimated to occur at a frequency at 1 in 10^5 to 10^6 with all vaccines. Should this occur resuscitation facilities and appropriately trained personnel will be available to manage the reaction.

Duty to assure high standards of informed consent

We will provide detailed information about the study for distribution to parents. The principal investigator will ensure that all parents fully understand the risks. As with any experimental vaccine the parents must understand that they have not yet been shown to prevent infection and this will be stressed during the recruitment stage. They must also understand the very small chance of anaphylactic reactions and thereby the importance of complying with the one hour observation period after each vaccination. The information sheet (attached) covers these points in detail, and each parent will have attended a public meeting, had the contents of the sheet explained in individual meetings on 2 separate occasions, including a quiz/ comprehension test administered prior to signing the form. This process is similar to that used for Phase 1 studies (SCC protocol no 650) and we believe has produced a high standard of informed consent.

Inducement

We would not offer any financial incentive to attend. There will be a perception of benefit from physical examination, laboratory screening and aspects of routine care such as treatment of iron deficiency and vitamin A supplementation. Health screening, and the early detection of malaria have been perceived as benefits in other studies.
We do not feel that these are benefits excessive, and all could be routine community based health care. We would also provide the children with toys and books during the time they are required to be observed in the clinic.

Animal subjects

Not applicable

12) Expected Application of Results

The current failure of control measures, the difficulty of providing and maintaining bed nets, the rise in drug resistance to commonly available antimalarials and the increase in malaria attributable mortality over recent years argue strongly the need for a malaria vaccine. Demonstrating efficacy against disease in the field is a critical step in the path for vaccine development. Furthermore, the study of immunogenicity and it's correlation with immunity will aid rational vaccine design to improve the product if this is required.

13) References

1. Herrington D, Davis J, Nardin E, et al. Successful immunization of humans with irradiated malaria sporozoites: humoral and cellular responses of the protected individuals. *Am J Trop Med Hyg* 1991;**45**(5)**:**539-47.
2. Mellouk S, Lunel F, Sedegah M, Beaudoin RL, Druilhe P. Protection against malaria induced by irradiated sporozoites. *Lancet* 1990;**335**(8691)**:**721.

3. Weiss WR, Mellouk S, Houghten RA, et al. Cytotoxic T cells recognize a peptide from the circumsporozoite protein on malaria-infected hepatocytes. *J Exp Med* 1990;**171**(3)**:**763-73.
4. Doolan DL, Hoffman SL. Pre-erythrocytic-stage immune effector mechanisms in Plasmodium spp. infections. *Philos Trans R Soc Lond B Biol Sci* 1997;**352**(1359)**:**1361-7.
5. Hill AV, Allsopp CE, Kwiatkowski D, et al. Common west African HLA antigens are associated with protection from severe malaria. *Nature* 1991;**352**(6336)**:**595-600.
6. Hill AV, Elvin J, Willis AC, et al. Molecular analysis of the association of HLA-B53 and resistance to severe malaria. *Nature* 1992;**360**(6403)**:**434-9.
7. Hill AV. The immunogenetics of resistance to malaria. *Proc Assoc Am Physicians* 1999;**111**(4)**:**272-7.
8. Flanagan KL, Lee EA, Gravenor MB, et al. Unique T cell effector functions elicited by Plasmodium falciparum epitopes in malaria-exposed Africans tested by three T cell assays. *J Immunol* 2001;**167**(8)**:**4729-37.
9. Flanagan KL, Mwangi T, Plebanski M, et al. Ex vivo interferon-gamma immune response to thrombospondin-related adhesive protein in coastal Kenyans: longevity and risk of Plasmodium falciparum infection. *Am J Trop Med Hyg* 2003;**68**(4)**:**421-30.
10. Reece WH, Pinder M, Gothard PK, et al. A CD4(+) T-cell immune response to a conserved epitope in the circumsporozoite protein correlates with protection from natural Plasmodium falciparum infection and disease. *Nat Med* 2004;**10**(4)**:**406-10.
11. Sun P, Schwenk R, White K, et al. Protective immunity induced with malaria vaccine, RTS,S, is linked to Plasmodium falciparum circumsporozoite protein-specific CD4+ and CD8+ T cells producing IFN-gamma. *J Immunol* 2003;**171**(12)**:**6961-7.
12. Robson KJ, Hall JR, Jennings MW, et al. A highly conserved amino-acid sequence in thrombospondin, properdin and in proteins from sporozoites and blood stages of a human malaria parasite. *Nature* 1988;**335**(6185)**:**79-82.
13. Schneider J, Gilbert SC, Blanchard TJ, et al. Enhanced immunogenicity for CD8+ T cell induction and complete protective efficacy of malaria DNA vaccination by boosting with modified vaccinia virus Ankara. *Nat Med* 1998;**4**(4)**:**397-402.
14. Schneider J, Langermans JA, Gilbert SC, et al. A prime-boost immunisation regimen using DNA followed by recombinant modified vaccinia virus Ankara induces strong cellular immune responses against the Plasmodium falciparum TRAP antigen in chimpanzees. *Vaccine* 2001;**19**(32)**:**4595-602.
15. McConkey S RW, Moorthy V, Webster D, Dunachie S, Gilbert S, Hill A V S. Enhanced T-cell immunogenicity of plasmid DNA vaccines boosted by recombinant modified vaccinia virus Ankara in humans. *nature medicine* 2003.
16. Webster D HAea. Efficacy and safety of prime boost immunisation using attenuated Fowlpox and MVA recombinant for the antigen ME-TRAP. *In preparation* 2003.
17. Bejon P, Andrews L, Andersen R, Dunachie S, Webster D, Walther M, Gilbert S, Peto T, Hill AVS. Calculation of Liver-to-Blood Inocula, Parasite Growth Rates and Pre-Erythrocytic Vaccine Efficacy from Serial Quantitative PCR Studies of Malaria Sporozoite Challengees. *Journal of Infectious Disease* In press.
18. Gurvich EB. The age-dependent risk of postvaccination complications in vaccinees with smallpox vaccine. *Vaccine* 1992;**10**(2)**:**96-7.
19. Mayr A, Stickl H, Muller HK, Danner K, Singer H. [The smallpox vaccination strain MVA: marker, genetic structure, experience gained with the parenteral vaccination and behavior in organisms with a debilitated defence mechanism (author's transl)]. *Zentralbl Bakteriol [B]* 1978;**167**(5-6)**:**375-90.

20. Mayr A, Baljer G, Wagner C, Sailer J. [Development of a numerically additive combined vaccine against tetanus and smallpox]. *Zentralbl Bakteriol Mikrobiol Hyg [A]* 1985;**259**(2)**:**206-18.
21. Moorthy V, Pinder, M., Watkins K, Atabani S, Bojang K, McAdam, J., Hill AVS,. Safety of DNA and MVA vaccines against liver-stage P. falciparum malaria. *in press* 2003.
22. Moorthy VS, Pinder M, Reece WH, et al. Safety and immunogenicity of DNA/modified vaccinia virus ankara malaria vaccination in African adults. *J Infect Dis* 2003;**188**(8)**:**1239-44.
23. Moorthy VS, McConkey S, Roberts M, et al. Safety of DNA and modified vaccinia virus Ankara vaccines against liver-stage P. falciparum malaria in non-immune volunteers. *Vaccine* 2003;**21**(17-18)**:**1995-2002.
24. Taylor J, Weinberg R, Languet B, Desmettre P, Paoletti E. Recombinant fowlpox virus inducing protective immunity in non-avian species. *Vaccine* 1988;**6**(6)**:**497-503.
25. Taylor J, Paoletti E. Fowlpox virus as a vector in non-avian species. *Vaccine* 1988;**6**(6)**:**466-8.
26. Laidlaw SM, Skinner MA. Comparison of the genome sequence of FP9, an attenuated, tissue culture-adapted European strain of Fowlpox virus, with those of virulent American and European viruses. *J Gen Virol* 2004;**85**(Pt 2)**:**305-22.
27. Rosenberg SA, Yang JC, Schwartzentruber DJ, et al. Recombinant fowlpox viruses encoding the anchor-modified gp100 melanoma antigen can generate antitumor immune responses in patients with metastatic melanoma. *Clin Cancer Res* 2003;**9**(8)**:**2973-80.
28. Cavacini LA, Duval M, Eder JP, Posner MR. Evidence of determinant spreading in the antibody responses to prostate cell surface antigens in patients immunized with prostate-specific antigen. *Clin Cancer Res* 2002;**8**(2)**:**368-73.
29. Moorthy V. Safety and immunogenicity of Fowlpox and MVA encoding TRAP in the Gambia. *In preparation* 2003.
30. Plebanski M, Gilbert SC, Schneider J, et al. Protection from Plasmodium berghei infection by priming and boosting T cells to a single class I-restricted epitope with recombinant carriers suitable for human use. *Eur J Immunol* 1998;**28**(12)**:**4345-55.
31. Schofield L, Villaquiran J, Ferreira A, Schellekens H, Nussenzweig R, Nussenzweig V. Gamma interferon, CD8+ T cells and antibodies required for immunity to malaria sporozoites. *Nature* 1987;**330**(6149)**:**664-6.
32. Bavarian-Nordic. Bavarian Nordic's HIV Vaccine Candidate Reveals Strong Saftey and Immunogenicity Profile in HIV Infected Patients. Copenhagen: Baverian-Nordic, 2002.
33. Xia Jin MR, Shady Barsoum, Geoffrey Deschenes, Lei Ba, James Binley, Daryl Schiller, Daniel bauer, David Ho, Martin Markowitz. Saftey and Immunogencitiy of ALVAC vCP1452 and recombinant gp160 in Newly Human Immunodeficiency Virus Type 1 infected patients treated with prolonged Highly Active Antiretroviral Therapy. *Journal of Virology* 2002;**76**(5)**:**2206-2216.
34. Alonso PL, Smith T, Schellenberg JR, et al. Randomised trial of efficacy of SPf66 vaccine against Plasmodium falciparum malaria in children in southern Tanzania. *Lancet* 1994;**344**(8931)**:**1175-81.
35. D'Alessandro U, Leach A, Drakeley CJ, et al. Efficacy trial of malaria vaccine SPf66 in Gambian infants. *Lancet* 1995;**346**(8973)**:**462-7.
36. Genton B, Betuela I, Felger I, et al. A recombinant blood-stage malaria vaccine reduces Plasmodium falciparum density and exerts selective pressure on parasite populations in a phase 1-2b trial in Papua New Guinea. *J Infect Dis* 2002;**185**(6)**:**820-7.

37. Nookala S, Srinivasan S, Kaliraj P, Narayanan RB, Nutman TB. Impairment of tetanus-specific cellular and humoral responses following tetanus vaccination in human lymphatic filariasis. *Infect Immun* 2004;**72**(5)**:**2598-604.
38. Cooper PJ, Espinel I, Paredes W, Guderian RH, Nutman TB. Impaired tetanus-specific cellular and humoral responses following tetanus vaccination in human onchocerciasis: a possible role for interleukin-10. *J Infect Dis* 1998;**178**(4)**:**1133-8.
39. Sabin EA, Araujo MI, Carvalho EM, Pearce EJ. Impairment of tetanus toxoid-specific Th1-like immune responses in humans infected with Schistosoma mansoni. *J Infect Dis* 1996;**173**(1)**:**269-72.
40. Malhotra I, Mungai P, Wamachi A, et al. Helminth- and Bacillus Calmette-Guerin-induced immunity in children sensitized in utero to filariasis and schistosomiasis. *J Immunol* 1999;**162**(11)**:**6843-8.
41. Armstrong Schellenberg JR, Smith, Alonso, Hayes. What is clinical Malaria? Finding case definitions for field research in highly endemic areas. *Parasitology Today* 1994;**10**(11)**:**439-441.
42. Baird JK, Owusu Agyei S, Utz GC, et al. Seasonal malaria attack rates in infants and young children in northern Ghana. *Am J Trop Med Hyg* 2002;**66**(3)**:**280-6.
43. Owusu-Agyei S, Koram KA, Baird JK, et al. Incidence of symptomatic and asymptomatic Plasmodium falciparum infection following curative therapy in adult residents of northern Ghana. *Am J Trop Med Hyg* 2001;**65**(3)**:**197-203.
44. Appawu M, Owusu-Agyei S, Dadzie S, et al. Malaria transmission dynamics at a site in northern Ghana proposed for testing malaria vaccines. *Trop Med Int Health* 2004;**9**(1)**:**164-70.
45. Smith T, Killeen G, Lengeler C, Tanner M. Relationships between the outcome of Plasmodium falciparum infection and the intensity of transmission in Africa. *Am J Trop Med Hyg* 2004;**71**(2 Suppl)**:**80-6.
46. Bojang KA, Milligan PJ, Pinder M, et al. Efficacy of RTS,S/AS02 malaria vaccine against Plasmodium falciparum infection in semi-immune adult men in The Gambia: a randomised trial. *Lancet* 2001;**358**(9297)**:**1927-34.
47. John CC, O'Donnell RA, Sumba PO, et al. Evidence that invasion-inhibitory antibodies specific for the 19-kDa fragment of merozoite surface protein-1 (MSP-1 19) can play a protective role against blood-stage Plasmodium falciparum infection in individuals in a malaria endemic area of Africa. *J Immunol* 2004;**173**(1)**:**666-72.
48. Sagara I, Sangare D, Dolo G, et al. A high malaria reinfection rate in children and young adults living under a low entomological inoculation rate in a periurban area of Bamako, Mali. *Am J Trop Med Hyg* 2002;**66**(3)**:**310-3.
49. Lengeler C. Insecticide-treated bednets and curtains for preventing malaria (Cochrane Review). *The Cochrane Library* 2004(1).
50. Stoute JA, Kester KE, Krzych U, et al. Long-term efficacy and immune responses following immunization with the RTS,S malaria vaccine. *J Infect Dis* 1998;**178**(4)**:**1139-44.
51. Looareesuwan S, Wilairatana P, Viravan C, Vanijanonta S, Pitisuttithum P, Kyle DE. Open randomized trial of oral artemether alone and a sequential combination with mefloquine for acute uncomplicated falciparum malaria. *Am J Trop Med Hyg* 1997;**56**(6)**:**613-7.
52. White NJ, Olliaro P. Artemisinin and derivatives in the treatment of uncomplicated malaria. *Med Trop (Mars)* 1998;**58**(3 Suppl)**:**54-6.

14) Budget

	USD	KSh

a) Personnel, salaries and benefits disbursement		200,000	14,000,000
b) Patient costs, travel, food and/or supplies		1,000	70,000
c) Equipment		10,000	700,000
d) Supplies		1,000	70,000
e) Travel and accommodation	- local	1,000	70,000
	- international	1,000	70,000
f) Transportation, vehicle repairs etc		25,000	1,750,000
g) Operating expenses postage, printing etc.		2,000	140,000
h) Animals acquisition etc.		0	
i) Consultancy fees		0	
j) Contingency fees (15% of above)		36,150	2,530,500
k) Institutional administrative overheads		0	
**Total**		277,150	19,400,500

15) Justification of the Budget

The study is part of the KEMRI-Wellcome Trust programme and will not incur any consultancy fees or additional administrative overheads. Costs for transport and field supplies are based on those incurred by projects of a similar size run in KEMRI-CGMRC. This will require 10 field workers, 10 motorbikes and running costs for a landrover.

16) Appendices

a) <u>State the role of each participating investigator</u>

PB: Community sensitisation, Study design, Day to day running and clinical responsibilities, Immunology.
TM: Project Manager, Data management
TL: Co-ordination of trial, monitoring, GCP compliance.
JM and OK: Immunology assays.
SM: Community sensitisation, communication material.
GF and PM: Statistical analysis
BL: Laboratory management, GLP compliance.
NP: Study Design and Overall co-ordination.
KM: Study Design and Overall co-ordination
AVSH: Study Design and Overall co-ordination

b) Please see attached documents for CV (for AVSH, TL) and Information Sheet/ Consent Form

c) Abbreviations used in the text;

ALT: Alanine Transaminase, a liver enzyme measured in serum to assess liver damage.

AMA: Apical Merozoite Antigen, a blood stage protein of the malaria parasite.

CRF: Case Report Form.

DNA:ME-TRAP: DNA encoding ME-TRAP

ELISpot: a technique for counting the number of T cells producing interferon gamma after *in vitro* stimulation.

FP9: An attenuated Fowlpox virus strain.

FP9:ME-TRAP : FP9 recombinant for ME-TRAP

HLA-B*53: a class I major histocompatability complex allele, present on most cells including hepatocytes, responsible for presenting antigen to cytotoxic T cells.

IFN gamma: interferon gamma is produced by activated T cells in response to antigen.

MHC: Major Histocompatability Complex

MSP 1: Merozoite Surface Protein 1.

MVA: Modified Vaccinia virus Ankara, an attenuated vaccinia virus strain.

MVA:ME-TRAP : MVA recombinant for ME-TRAP

ME-TRAP: the combination of a string of multiple epitopes from *Plasmodium falciparum* pre-erythrocytic antigens with the whole of the pre-erythrocytic antigen Thrombospondin Related Adhesion Protein.

RTS,S: a particulate vaccine fusing some of the circumsporozoite protein with hepatitis B surface antigen.
