# Supplementary material for: A Phase 2b Randomised Trial of the Candidate Malaria Vaccines FP9 ME-TRAP and MVA ME-TRAP among Children in Kenya
Source: PLoS Clin Trials. 2006 Oct 20;1(6):e29. doi: 10.1371/journal.pctr.0010029 (PMC1617125; doi:10.1371/journal.pctr.0010029)
Supplement: Alternative Language Abstract S1 — (23 KB DOC) [file pctr.0010029.sd003.doc]

Kifupi

Malengo

Malengo yalikuwa kupima chanjo zitwazo “FFM ME-TRAP” zikifanya kazi kukinga kutokana malaria kati ya watoto kwenye malaria. “FFM ME-TRAP” ni kuchanja na virusi viwili vilivyosinziwa mbili (FP9 na ndui iliyosinziwa kutoka Ankara), kutoa sehemu za malaria ziitwazo ME-TRAP.

Shauri

Chanjo ilipimwa kati ya majaribio yaliyobahatishwa, na yaliyolinganishwa na chanjo ingine.

Pahali

Majaribio yalifanyika kwenye pwani ya Kenya, palipo na malaria.

Washiriki

Washiriki walikuwa 405 watoto wenye afya, kutoka mwaka moja mpaka miaka sita.

Uingiliaji

Washiriki walibahatishwa kuchanjwa na FFM ME-TRAP au chanjo ya kichwa cha mbwa.

Kipimo

Baada ya kupewa dawa ya malaria, watoto walifuatilizwa kwa muda ya miezi tisa. Walipotembelwa kila wiki, joto la mwili ilipimwa. Damu iliangaliwa kwa watoto wenye moto. Kipimo cha kwanza kilkuwa wakati mpaka kupata malaria (>2,500/ μl) na moto.

Majibu

Chanjo iliongeza nguvu ya damu kidogo, lakini si kama utafiti wa awali. Kufikiria watoto wote waliobahatishwa kupewa chanjo, watato wenye homa ya malaria walikuwa wengi kati ya wenye FFM ME-TRAP kuliko wasiyochanjwa na FFM ME-TRAP (52/190 vs 40/197). Uwiano ilikuwa 1.52, lakini inaweza kuwa kwa sababu ya bahati mbaya peke yake (95% CI 1.0-2.3, P=0.14 kwa logrank). Watoto 346 walichanjwa kulingana na mipango. Kwa hawa watoto, uwiano ilikuwa 1.3 (95% CI 0.8-2.1, p=0.55).

Kufikiria watoto waliopata malaria mara nyingi, uwiano ilikuwa 1.3 (95% CI 0.8 mpaka 2.1, p=0.55).

Viwango vya damu ya watoto na kuwa na malaria bila homa, kwa miezi mitatu na miezi tisa baada ya kudungwa, vilkuwa si tofauti kwa wenye chanjo. Kati ya wenye chanjo FFM ME-TRAP, nguvu ya damu iliyoongezeka na chanjo, haiunganishi na kupata malaria.

Hatima

Chanjo haikingi kutokana na malaria na homa. Utafiti ujao utahitaji chanjo zenye nguvu zaidi zitakopotumika kwa watoto wanaoishi kwenye malaria.
