# Supplementary material for: A Phase 2b Randomised Trial of the Candidate Malaria Vaccines FP9 ME-TRAP and MVA ME-TRAP among Children in Kenya
Source: PLoS Clin Trials. 2006 Oct 20;1(6):e29. doi: 10.1371/journal.pctr.0010029 (PMC1617125; doi:10.1371/journal.pctr.0010029)
Supplement: Text S1 — (37 KB DOC) [file pctr.0010029.sd004.doc]

VAC 32: Safety, immunogenicity and efficacy against febrile malaria of the candidate vaccines FP9 ME-TRAP and MVA ME-TRAP for children in an endemic area.

Analysis Plan, Version 3. 9^th^ February 2006

P. Bejon

Timeline.

Last trial observation; Late January 2006

Data Entry complete; Mid February 2006

Query and Data cleaning Early March 2006

Unblinding Mid March 2006

Data transmitted to DSMB and archived in Kilifi Mid March 2006

Analysis complete and discussed by investigators Mid March 2006

Final report to DSMB and sponsor April 2006

Analysis populations

1. Adverse events will be analysed by all subjects who received at least one vaccination, irrespective of protocol violations, or administration of the alternate vaccine, rather than the one indicated by randomisation. This will Include everyone who got at least one dose. Subjects not receiving second or third vaccinations will contribute adverse event data for the first vaccination only. Subjects who received a sequence of vaccinations that included an error of vaccine allocation will be tabulated separately.
2. Immunogenicity will be analysed by all subjects who received correct vaccination at the correct times (i.e. at 15-35 day intervals). Incorrect vaccinations will be excluded from this group.
3. Efficacy will be analysed by intention to treat (ITT); i.e. everyone randomised with follow up data (at least one visit), and then according to protocol (ATP), ie restricted to those receiving full and correct vaccinations, who were followed up at least once.

Database handling and unblinding

Data is currently stored in studybuilder format i.e. several "long" or "flat" tables divided by time rather than wide tables divided by variable category. There are two other sources of data; a key, linking vaccination allocation cards to study subject numbers, and paper cards prepared according to that key, which the study nurses used make a written record of vaccination.

The studybuilder, source documents will be converted to conventional formats using stata in kilifi. Data will be compared with the original studybuilder files to confirm faithful conversion and a preliminary, blinded, analysis performed to identify and resolve queries. The cleaned data will then be viewed by Paul Milligan, who will conduct a second, blind, preliminary analysis. Final queries will be resolved at this stage. When it is confirmed that a complete and consistent data set has been received the data base (with the exception of the vaccination records) will be locked and vaccination allocation will be unblinded. Vaccination records will then be inspected by the investigators, and any discrepancies resolved by examining the concurrent paper records. Discrepancies will be noted and tabulated. A copy of the final database will be sent to the DSMB before further analysis.

Description

A trial profile will be produced; including numbers of subjects screened, randomized, receiving each vaccine correctly, the numbers completing follow up and a description of protocol violations; including receipt of all scheduled vaccines, correct vaccine type, dose and interval between doses, receipt of anti malarial drugs and clearance of parasites before follow up.

Reasons for subject withdrawal will be tabulated by time.

Adverse events

**Severe adverse events** will be individually described by the investigators.

**Solicited adverse events** for each day (days 1,2, 3 and 7 post vaccination), will be tabulated; recording overall frequency of event, and frequency for each grade of intensity. Where intensity is described by a continuous variable the mean will be given. In these cases the single highest diameter seen during the post vaccination week will be taken for each subject. First, second and third immunizations will be examined separately comparing active vaccine with placebo.

**Unsolicited adverse events** will be assigned categories by the investigators before the data is locked and tabulated by vaccination and by three different time periods (ie in the first week, in the first month or at any time during the year s follow up )

Median values and non parametric tests will be used to compare full blood counts, creatinine and ALT measurements between vaccine groups.

Immunogenicity

Background (cells cultured without peptide) will be subtracted from peptide provoked responses. These will be summed, to derive totals for each individual. An appropriate transformation will be used to normalise data, and cultured and ex vivo ELISpots at each of the 3 post vaccination timepoints compared by general linear models.

Assays would be discarded unless the control well (PHA) showed 100 spots or more and the negative control well less than 40 spots for ex vivo, or 80 spots for cultured ELISpots.

Further exploratory analysis will examine the effect of various baseline factors on immunogenicity. These will include being malaria positive at either screening or immediately post-vaccination, having an acute episode of malaria during the immunisation course, stool and urine microscopy for parasites, eosinophilia, age, nutrition (measured by MUAC) and prior BCG vaccination status.

Efficacy

The following primary analyses of efficacy will be conducted both According to Protocol and Intention to Treat. The Secondary analyses will be According to Protocol.

The primary endpoint for analysis will be febrile (or “mild”) malaria; defined as fever (axilliary temperature 37.5 degrees or higher) and greater than 2500 parasites per ul (as calculated from a thick or thin blood film using the WHO method), and secondarily fever with any parasitaemia (since parasitaemia had been cleared by treatment with artesunate before follow up). Mixed infections will be considered for the study endpoint, single infection with non falciparum species will not be considered.

Further secondary analysis will use the parasite density threshold most appropriate to define malaria in this population, using the logistic regression modelling described by Shellenberg and Smith (1996). It is unlikely this will be higher than 2500 parasites per ul; this threshold had been confirmed in the studied age group in different transmission areas in Kilifi (Mwangi, 2005).

An exploratory analysis will compare the thresholds estimated for the active versus placebo group.

For these analyses vaccine efficacy is defined as VE=1-R, where R is the hazard ratio (malaria vaccine group:rabies group) estimated using Cox's proportional hazards model including all the covariates (specify). A 95% confidence interval for VE and a P-value (from the likelihood ratio test) will be computed. Interaction effects between vaccine group and age, and vaccine group and bednet use, will be examined. Graphical methods will be used to examine the proportional hazards assumption. A Kaplan-Meier graph will be produced showing the number at risk at selected time points. Cumulative incidence of infection by the end of surveillance will be calculated for each group.

Primary Analysis: Survival analysis of time to infection

The primary analysis will be a log rank test for time to event (as defined above) by vaccine allocation, stratified by the following covariates; village (a factor with 5 levels) then being parasite positive before follow up and finally age at immunisation as a category (1-2 years old, 2-5 year and 5 or older). Efficacy within age categories will be a secondary endpoint.

Finally, log rank analysis would be conducted within three blocks of three months follow up, to derive estimates of efficacy in each time period. This will test how sustained protection was over time.

Cox regression for time to first event will then be performed. Vaccine allocation as a factor would initially be adjusted for by the same covariates; village (a factor with 5 levels), being parasite positive before follow up and age at immunisation as a category (1-2 years old, 2-5 year and 5 or older) and bednet use at baseline.

Bed net use has changed during the period of the trial. It will be analysed first as a time dependant covariate (since repeated surveys of use have been conducted); but if found to not be significantly associated with outcome (p<0.05), will be analysed as a single, time independent factor on the basis of the first survey. Bed net use is documented when the field worker confirms the bed net is hanging over the childs sleeping space, and has less than 5 holes able to fit three fingers. Bed nets have been distributed by the local dispensary, and were treated when distributed.

Other factors will be nutrition (categorized above and below median MUAC), eosinophilia, and the presence of intestinal worms. These will be discarded if not significant (p>0.05). Where children were not seen for 3 consecutive weeks they will be censored and not contribute to the population at risk, and where antimalarial use (we’ve given out) is documented (with or without a positive blood film) the subject will be censored for 4 weeks. If these are significantly associated with outcome, they will be included in the stratification for the logrank test and as covariates in the cox regression in the primary analysis.

Antimalarial use (in children without parasites by blood film) and frequency of non-malaria fever will be tabulated by vaccination group.

Immunogenicity will be assessed according to tertiles from both ex vivo and cultured ELISpot assays. Efficacy will be analysed among subjects receiving the malaria vaccine according to both factors. The tertiles will be compared with control vaccinations.

As a secondary analysis, if appropriate, estimates of efficacy will be adjusted for the effect of unmeasured covariates, to be fit as shared frailties with a gamma distribution.

Multiple Episodes

The distribution of episodes will be tested for over-dispersion, to guide choice of either negative binomial or poisson regression for the analysis. To allow for seasonal variation in malaria incidence, the number of episodes will be analysed by three blocks of 3 months; each categorized by time period and child. The same co-variates and potential factors will be examined. Bed net use will be specified for each 3 month period of monitoring. Both ex vivo and cultured ELISpot results will be used as continuous variables (log transformed, calculated as described under immunogenicity) from the blood test relevant to each monitoring period; ie. the first week, post vaccination test for the first 3 month monitoring, the test 3 months post vaccination for the second three months, and the test at the end of 9 months for the final three months. Events will be censored according to antimalarial use for four weeks after taking the medication. Variation in efficacy with time will be examined by testing for the interaction between vaccine group and time period.

Secondary analysis.

The impact of vaccination on haemoglobin at three months, and at 9 months (the end of follow up) will be compared by vaccination group; adjusting for haemoglobin at screening and at one week by linear regression.

The impact of vaccination on parasite prevalence during the cross-sectional bleeds conducted at 3 months and at 9 months will be measured, adjusting for co-variates as detailed above for primary analysis.

Finally an exploratory analysis would estimate of the Malaria Attributable Fever fraction within active and placebo groups will be made following the logistic regession model described above. This would assess the impact of curative treatment on the Attributable Fever Fraction by comparing the first and second half of the immunisation period.

Continued monitoring.

Continued surveillance for episodes of malaria will be conducted in the area after the end of January 2006. All immunised children will be invited to join this continued monitoring. The analysis plan described above (with the exception of solicited adverse events) will be repeated on a subsequent 9 months data, ending October 2006.
